# A Portable Droplet Magnetofluidic Device for Point-of-Care Detection of Multidrug-Resistant *Candida auris*

**DOI:** 10.3389/fbioe.2022.826694

**Published:** 2022-03-24

**Authors:** Pei-Wei Lee, Marissa Totten, Liben Chen, Fan-En Chen, Alexander Y. Trick, Kushagra Shah, Hoan Thanh Ngo, Mei Jin, Kuangwen Hsieh, Sean X. Zhang, Tza-Huei Wang

**Affiliations:** ^1^ Department of Mechanical Engineering, Johns Hopkins University, Baltimore, MD, United States; ^2^ Division of Microbiology, Department of Pathology, Johns Hopkins School of Medicine, Baltimore, MD, United States; ^3^ Department of Biomedical Engineering, Johns Hopkins University, Baltimore, MD, United States; ^4^ Institute of NanoBioTechnology, Johns Hopkins University, Baltimore, MD, United States

**Keywords:** droplet magnetofluidics, point-of-care diagnostics, sample preparation, lysis, miniaturized rapid PCR, *Candida auris*, multidrug resistance, fungal pathogen

## Abstract

*Candida auris* is an emerging multidrug-resistant fungal pathogen that can cause severe and deadly infections. To date, *C. auris* has spurred outbreaks in healthcare settings in thirty-three countries across five continents. To control and potentially prevent its spread, there is an urgent need for point-of-care (POC) diagnostics that can rapidly screen patients, close patient contacts, and surveil environmental sources. Droplet magnetofluidics (DM), which leverages nucleic acid-binding magnetic beads for realizing POC-amenable nucleic acid detection platforms, offers a promising solution. Herein, we report the first DM device—coined POC.auris—for POC detection of *C. auris*. As part of POC.auris, we have incorporated a handheld cell lysis module that lyses *C. auris* cells with 2 min hands-on time. Subsequently, within the palm-sized and automated DM device, *C. auris* and control DNA are magnetically extracted and purified by a motorized magnetic arm and finally amplified *via* a duplex real-time quantitative PCR assay by a miniaturized rapid PCR module and a miniaturized fluorescence detector—all in ≤30 min. For demonstration, we use POC.auris to detect *C. auris* isolates from 3 major clades, with no cross reactivity against other *Candida* species and a limit of detection of ∼300 colony forming units per mL. Taken together, POC.auris presents a potentially useful tool for combating *C. auris*.

## Introduction

Multidrug-resistant microorganisms present an ongoing global healthcare crisis. Among them, *Candida auris* is an emerging nosocomial fungal pathogen (Meis and Chowdhary, 2018; Vallabhaneni et al., 2019) that is resistant to antifungal drugs and can cause severe infections. Indeed, over 40% of *C. auris* isolates are resistant to 2 or more antifungal drug classes (Chowdhary et al., 2018; Kordalewska et al., 2018; Tsay et al., 2018; Hou et al., 2019; Park et al., 2019), while severe healthcare-associated invasive infections caused by *C. auris* may reach a mortality rate up to 60% in patients with underlying comorbidities (Schelenz et al., 2016; Eyre et al., 2018; Meis and Chowdhary, 2018; Armstrong et al., 2019). Importantly, *C. auris* acquisition appears most likely due to exogenous exposure with the healthcare environment being the major reservoir (Welsh et al., 2017; Sexton et al., 2018; Armstrong et al., 2019). *C. auris* can remain viable on medical devices, instrument, equipment, furniture, and beddings for up to 14 days at hospital settings (Welsh et al., 2017). *C. auris* may also colonize in patients without causing clinical symptoms for up to 3 months (Eyre et al., 2018; Bradley, 2019). The long viability in the hospital environment and persistent colonization in patients facilitate transmission and spread of the organism in healthcare settings, as evidenced by several nosocomial outbreaks in the United States and reported infections in thirty-three countries across five continents (Schelenz et al., 2016; Vallabhaneni et al., 2017; Adams et al., 2018; Eyre et al., 2018; Jeffery-Smith et al., 2018; Armstrong et al., 2019). Currently, control and prevention of the spread of *C. auris* requires monitoring patients, screening close patient contacts, and surveilling environmental sources for the organism (Adams et al., 2018; Eyre et al., 2018; Ku et al., 2018; Tsay et al., 2018). Effectiveness of this practice critically depends on reliable, rapid, cost-effective, and ideally, point-of-care (POC) identification of *C. auris*. Unfortunately, despite advances in laboratory-based methods that can identify *C. auris* (e.g., MALDI-TOF mass spectrometry (Mizusawa et al., 2017) and benchtop PCR (Kordalewska et al., 2017; Arastehfar et al., 2018; Leach et al., 2018; Sexton et al., 2018; Ahmad et al., 2019; Hou et al., 2019; Jainlabdin et al., 2019; Kordalewska et al., 2019; Leach et al., 2019; Lima et al., 2019)), an urgent but unmet need for POC identification of *C. auris* still remains.

Droplet magnetofluidics (DM), in recent years, has emerged as a promising technology for detecting pathogenic nucleic acids at POC (Zhang et al., 2011; Chiou et al., 2013; Zhang and Wang, 2013; Shin et al., 2014; Shin and Wang, 2014; Shin et al., 2017; Shin et al., 2018; Chen F.-E. et al., 2021; Chen L. et al., 2021; Trick Alexander et al., 2021). DM uses magnetic beads to capture nucleic acids and transport them (with the assistance of external magnets) between discrete droplets of reagents and achieve extraction, purification, and amplification of nucleic acids. Such use of magnetic beads facilitates automation and obviates fluidic control components such as pumps and valves for manipulating reagents, which significantly increases the amenability of DM-based devices toward POC use. More recently, small thermoplastic cartridges and fully integrated yet portable devices have been developed to perform DM-based pathogen detection (Shin et al., 2018; Chen F.-E. et al., 2021; Chen L. et al., 2021; Trick Alexander et al., 2021). In addition to automating DM-based nucleic acid extraction and purification, the extruded wells in the cartridges and the miniaturized thermocycling units and fluorescence detectors in the integrated devices facilitate ultrafast real-time quantitative PCR and enable rapid detection. It is, therefore, a promising opportunity to leverage the advent of DM technology toward developing POC detection of *C. auris*.

We are, thus, motivated to develop such a portable DM device for POC detection of *C. auris*—conveniently named POC.auris. As part of POC.auris, we incorporate a handheld cell lysis method that rapidly and effectively lyses *C. auris* cells. *C. auris* DNA in the lysate is extracted, purified, and amplified in full automation within the DM assay cartridge and the DM device. A duplex real-time quantitative PCR (qPCR) assay that specifically detects *C. auris* and an internal assay control plasmid is implemented in POC.auris. Importantly, when performed in the DM assay cartridge and the portable DM device, the duplex qPCR assay is miniaturized and as a result accelerated to ∼26 min. For demonstration, we use POC.auris to detect *C. auris* isolates from 3 major clades that originate from South Asia, Africa, and South America, with no cross reactivity against multiple non-*C. auris Candida* species and a limit of detection (LOD) of 300 colony-forming units (CFU)/mL.

## Materials and Methods

### *Candida* Species, Storage, Subculture, and Handling

All *Candida* species tested in this work were isolates from either the United States Center for Disease Control and Prevention or the Johns Hopkins Hospital Microbiology Laboratory (Supplementary Table S1). *Candida auris* (*C. auris*) AR0382 (a strain belonging to the South Asia clade), AR0384 (a strain belonging to the Africa clade), AR0385 (a strain belonging to the South America clade), and AR0388 (a strain belonging to the South Asia clade) were obtained from the United States Center for Disease Control and Prevention. *C. auris* Pt3 (i.e., Patient #3) was a clinical isolate (originating from India, which belongs to the South Asia clade) obtained from the Johns Hopkins Hospital Microbiology Laboratory. Other *Candida* species, including *C. albicans*, *C. duobushaemulonii*, *C. glabrata*, *C. haemulonii*, *C. krusei*, *C. parapsilosis*, and *C. tropicalis* were also clinical isolates from the Johns Hopkins Hospital Microbiology Laboratory and were identified to the species level *via* in-house MALDI-TOF mass spectrometry (MALDI Biotyper^®^, Bruker, Billerica, MA).

All isolates were kept at −80°C in Microbank™ 2D vials that are filled with beads and specially formulated cryopreservative. For subculture, a single bead was removed from Microbank™ 2D vial, plated on Sabouraud Dextrose Agar (SDA; pH 5.6), incubated at 30°C for 24—72 h, and observed periodically throughout incubation. If only a few isolates were observed after 72 h, then an isolate from the SDA plate was plated onto a new SDA plate and grown under the same conditions. For making cell suspensions and dilutions, colonies were scraped and suspended in phosphate-buffered saline (PBS). The cell suspension was measured *via* optical density at wavelength 600 nm (i.e., OD600) to estimate its concentration, where OD600 = 0.1 was approximated as 3 × 10^6^ CFU/ml (Bravo Ruiz et al., 2019; Lima et al., 2019; Srivastava and Ahmad, 2020). All *Candida* species were handled within biosafety cabinets and with appropriate personal protective equipment in accordance with the biosafety level 2 (BSL-2) guideline.

### Benchtop Extraction and Purification of Genomic DNA From *Candida* Species

Genomic DNA from all *Candida* cell suspensions were extracted and purified using Quick-DNA Fungal/Bacterial Miniprep Kit (Zymo Research, Irvine, CA), which is based on bead beating and spin column purification. Briefly, a pea sized amount of *Candida* cells was directly added to 750 µL BashingBead™ Buffer and lysed in a ZR BashingBead™ Lysis Tube (0.1 and 0.5 mm), on a vortexer fitted with a bead-beating adapter at maximum speed for 10 min, and finally spun down. The supernatant was subsequently filtered through a Zymo-Spin™ III-F Filter *via* centrifugation. The filtrate was mixed with the Genomic Lysis Buffer (without beta-mercaptoethanol) and transferred to a Zymo-Spin™ IICR Column, where binding, washing, and elution of DNA were performed. The elution volume was 100 µL for all samples. Finally, the DNA concentrations for all samples were quantified using a Qubit fluorometer (Invitrogen, Waltham, MA).

### Preparation of Control DNA Plasmid

Two versions of the control DNA plasmid, both of which contain a fragment of the bicoid gene (GenBank Accession Number: NT_033777.3, Position: 6755842—6759466), were used in this work. One version of the plasmid was acquired from Addgene (Plasmid # 34340; Watertown, MA) as *E. coli* in an agar stab culture. This plasmid is named Addgene plasmid hereafter. Addgene plasmid-containing *E. coli* colonies were selected by plating the agar stab culture on agar plates containing kanamycin (50 mg/ml) and incubating at 37°C overnight. Subsequently, Addgene plasmid from plasmid-containing *E. coli* colonies were purified from *E. coli* using ZymoPure II Plasmid Maxiprep kit (Zymo Research, Irvine, CA) according to the manufacturer’s specifications, with the exception of the *E. coli* colonies that were directly suspended in the ZymoPURE P1 solution to begin the purification process. Unused plasmid-containing *E. coli* colonies from the kanamycin-containing agar plates were transferred to Microbank™ 2D vials and stored at −80°C. Once extracted, Addgene plasmid was aliquoted into microcentrifuge tubes and stored at −20°C. The other version of the plasmid was obtained as a gBlock from Integrated DNA Technologies (IDT; Coralville, IA). This plasmid is named IDT plasmid hereafter. The IDT plasmid was resuspended in UltraPure™ DNase/RNase-free distilled water (Thermo Fisher Scientific, Waltham, MA) at 100 µM concentration, aliquoted into 0.5 ml DNA Lo-Bind tubes (Eppendorf, Germany), and stored at −20°C.

### Benchtop Droplet Magnetofluidic-Compatible Rapid Duplex qPCR

The rapid duplex qPCR assay employed in this work was adopted and slightly modified from a previously reported assay (Leach et al., 2018) that had been adopted by the United States Center for Disease Control and Prevention. The duplex qPCR assay targets both *C. auris* and an assay control. The *C. auris* PCR primers and FAM-labeled TaqMan probe (Table 1; purchased from IDT) target a region within ITS2 gene that is specific to *C. auris*. The assay control PCR primers and TYE-labeled Taqman probe (Table 1; purchased from IDT) target a region within the bicoid gene (GenBank Accession Number: NT_033777.3, Position: 6755842—6759466). Lyophilized PCR primers and TaqMan probes were reconstituted in nuclease-free water (Promega, Madison, WI) at 100 µM. Reconstituted oligonucleotides were stored at −20°C. Promega GoTaq Probe qPCR Master Mix (Promega, Madison, WI) was used for all PCR reactions throughout this work. Unless otherwise specified, a typical benchtop rapid duplex qPCR assay (20 µL) included 1× PCR mix, 500 nM of *C. auris* forward and reverse primer each, 100 nM of *C. auris* probe, 100 nM of bicoid forward and reverse primer each, 100 nM of bicoid probe, 1 mg/ml BSA (New England BioLabs, Ipswich, MA), 0.05% Tween 20 (Millipore-Sigma, St. Louis, MO), 2 µL bicoid plasmid (1 pg for IDT plasmid or 10 pg for Addgene plasmid), and 1 µL genomic DNA from *Candida* species (various amount for each reaction, either bench top extracted and purified or magnetically purified and eluted into the PCR mix). All PCR assays sans bicoid plasmid and genomic DNA from *Candida* species were prepared inside a PCR hood (AirClean Systems, Creedmoor, NC). Bicoid plasmid and genomic DNA from *Candida* species were added inside a separate biosafety cabinet (The Baker Company, Sanford, ME) to minimize contamination. All benchtop PCR reactions were performed in a Bio-Rad CFX96 Touch Real-Time PCR Detection System (Bio-Rad, Hercules, CA). Unless otherwise specified, a typical benchtop PCR reaction began with a 95°C hot start for 2 min, followed by 40 cycles of 95°C for 3 s and 60°C for 15 s, during which the fluorescence signals were measured every cycle. The fluorescence signals measured by the Bio-Rad CFX96 system were baseline subtracted *via* the built-in curve fit function in the CFX Manager Software and the PCR cycles of quantification (C_q_) were determined by the built-in single threshold algorithm in the CFX Manager Software.

**TABLE 1:** Primer and probe sequences for detecting the *C. auris* ITS2 region and control plasmid containing the bicoid gene.

Name	Sequence (5′—3′)
C_AUR_ITS2_F	CAG​ACG​TGA​ATC​ATC​GAA​TCT
C_AUR_ITS2_R	TTT​CGT​GCA​AGC​TGT​AAT​TT
C_AUR_ITS2_P	/6-FAM/AATCTTCGC/ZEN/GGTGGCGTTGCATTCA/IABkFQ/
BICOID_F	CAGCTTGCAGACTCTTAG
BICOID_R	GAATGACTCGCTGTAGTG
BICOID_P	/TYE665/AACGCTTTGACTCCGTCACCCA/IAbRQSp/

Manual magnetic-based DNA purification was added to complete the development of benchtop droplet magnetofluidic-compatible rapid duplex qPCR assay. ChargeSwitch™ gDNA Plant Kit (CS18000, Thermo Fisher Scientific, Waltham, MA), which can isolate genomic DNA from fungal samples, was used in this work. 1 µL *C. auris* genomic DNA, 1 µL control DNA plasmid and 1 µL of 3.6 μg/ml human genomic DNA (Promega, Madison, WI) were added to 97 µL 1× PBS (Thermo Fisher Scientific, Waltham, MA) to make the 100 µL sample. The magnetic bead buffer in this work was made by mixing 4 µL of ChargeSwitch magnetic particles (25 mg/ml in 1 mM sodium acetate, pH 4.5), 10 µL Binding Buffer from ChargeSwitch™ gDNA Plant Kit and 8 µL 10% Tween 20. The 100 µL sample was pipette mixed with the 22 µL magnetic bead buffer in a 1.5 ml microcentrifuge tube. The mixture was kept at room temperature for 1 min to allow binding between magnetic beads and *C. auris* DNA and control DNA plasmid. The tube was placed onto a DynaMag™-2 Magnet (Thermo Fisher Scientific, Waltham, MA) until a visible pellet of magnetic beads was formed on the tube wall before the supernatant was removed *via* pipetting. Forty nine µL wash buffer from ChargeSwitch™ gDNA Plant Kit with 1 µL 10% Tween 20 was subsequently added into the tube to wash the magnetic beads. The tube was briefly centrifuged and again placed onto the DynaMag™-2 Magnet to pellet the magnetic beads and remove the supernatant. Next, 10 µL rapid duplex qPCR mix was added to the pelleted magnetic beads in the tube. The tube was briefly centrifuged , kept at 80°C for 2 min to elute DNA into the PCR mix, and again placed onto the DynaMag™-2 Magnet to pellet and separate the magnetic beads from the PCR mix. The PCR mix (along with eluted DNA) was pipette transferred into a PCR tube (0.2 ml 8-Tube PCR Strips, Bio-Rad, Hercules, CA) before commencing PCR in the Bio-Rad CFX96 Touch Real-Time PCR Detection System.

### Droplet Magnetofluidic Assay Cartridge Fabrication

The design and fabrication of the DM assay cartridge, in which the POC.auris assay was performed, were based on our previous works (Chen F.-E. et al., 2021; Chen L. et al., 2021; Trick Alexander et al., 2021). Briefly, the assay cartridge, which measures approximately 4 cm (L) × 1 cm (W) × 1 cm (H) and houses a sample well, a wash buffer well, and a PCR mix well, was fabricated from inexpensive plastic components (material cost per assay cartridge = USD $0.31, Supplementary Table S2) *via* laser-cutting and thermoforming. The assay cartridge was composed of 3 layers: a top cap layer for establishing a sample injection opening while enclosing the rest of the assay cartridge, a center spacer layer for joining the layers, and a bottom well layer for holding the assay reagents. The cap layer of each assay cartridge was laser-cut by a CO_2_ laser-cutter from a polymethylmethacrylate (PMMA) sheet laminated with a polytetrafluoroethylene (PTFE) tape (McMaster-Carr, Elmhurst, IL, USA). Except for the inlet opening that was laser-cut into the cap layer, the rest of cap layer fit atop the spacer layer to enclose the assay cartridge. The spacer layer was laser-cut by the CO_2_ laser-cutter from a PMMA sheet laminated with pressure-adhesive tape (PSA, 9472LE adhesive transfer tape, 3M, USA) on both sides. The well layer was thermoformed from 0.2-mm-thick polypropylene sheet (AKAHA) over three-dimensional (3D)-printed molds (Form 2, Formlabs, Somerville, MA, USA) designed in Solidworks 2017 computer-aided design (CAD) software (Dassault Systèmes, Vélizy-Villacoublay, France) using a commercial dental vacuum forming instrument (Meta Dental Corp, Glendale, NY) to produce extruded wells. Upon fabrication of these individual layers, the spacer layer and the well layer were first assembled *via* PSA into an open assay cartridge. Both the cap layer and the open assay cartridge were kept separately at room temperature until full assembly for experimentation.

### Droplet Magnetofluidic Device Design and Construction

The design and assembly of the integrated portable DM device for automating POC.auris were based on our previous works (Chen L. et al., 2021; Trick Alexander et al., 2021). Briefly, the device was composed of a motorized magnetic arm, a miniature thermocycling module, a fluorescence detector (Fluo Sens Integrated, Qiagen), and a microcontroller (Arduino Uno R3) with a motorshield (Arduino Motor Shield Rev3) and a custom printed circuit board shield. The motorized magnetic arm was a 3D-printed (Formlabs Form 2) shaft with a pair of permanent neodymium magnets that were actuated by a rotational servo motor (HS–485HB Hitec RCD, Poway, CA, USA) and a linear servo motor (PQ12-R Actuonix, Victoria, BC, Canada). The bi-axial movement of the motorized magnetic arm pulled the beads in and out of each well, as well as across different wells of the DM cartridge, thereby achieving magnetic transfer within the cartridge. The miniature thermocycling module is the assembly of an aluminum heat block, a thermoelectric cooler, a heat sink, and a miniature fan. The fluorescence detector, which can detect both green fluorescence (excitation wavelength at 470 nm and emission wavelength at 520 nm) and red fluorescence (excitation wavelength at 625 nm and emission wavelength at 680 nm), was positioned and aligned to the PCR well of the cartridge at a predetermined distance. The microcontroller controlled the other 3 components—the movement of the motorized magnetic arm, the temperatures of the miniature thermocycling module (with electrical current supplied by the motorshield), and the timing for acquiring fluorescence signals by the fluorescence detector. The motorized magnetic arm, the fluorescence detector, and the microcontroller were affixed within the main housing of the device, whereas the miniature thermocycling module was affixed on the detachable faceplate, both of which were 3D-printed (Formlabs Form 2, black resin). The parts were embedded with permanent neodymium magnets, such that they could be aligned and magnetically clasped for attachment. The material cost of the DM device used in this work—a one-time cost—is USD $3539.41, nearly 90% of which is attributed to the fluorescence detector (Supplementary Table S3).

### Handheld Lysis of *C. auris*

In this work, handheld lysis of *C. auris* was achieved by using a commercial OmniLyse device (Claremont BioSolutions, Upland, CA), which performs micro-motor-based bead-beating mechanical lysis. Importantly, all handheld lysis of *C. auris* was performed within a biosafety cabinet and with appropriate personal protective equipments in accordance with the BSL-2 guideline. For performing lysis, the single-use lysis module was first connected to a single-use 5 ml syringe and attached to the reusable Bat-Pac™ battery pack *via* their electrical connectors. Immediately before loading a cell suspension into the lysis module, it was pre-washed by passing 500 µL1× PBS back and forth through it five times while it was switched on. After pre-washing, the module was emptied and switched off. *C. auris* cells (and other *Candida* species cells) were suspended in either 1× PBS or ESwab^®^ medium (Copan Diagnostics, Murrieta, CA) to a final volume of 500 µL in a 1.5 ml microcentrifuge tube (DNA Lo-Bind, Eppendorf, Germany). For one cycle of lysis, the cell suspension was drawn through the powered-on lysis module into the syringe and infused through the powered-on lysis module into the microcentrifuge tube in ∼5—10 s. Unless otherwise specified, ten cycles were performed, which could be accomplished in <2 min. After lysis, the lysate was entirely infused into the microcentrifuge tube and subsequently pipette loaded into the sample well of the droplet magnetofluidic assay cartridge to initiate POC.auris. The used lysis module and the used syringe were discarded. Of note, each lysis module costs USD $11.25, which currently represents the most expensive component of the POC.auris assay (Supplementary Table S4).

### POC.auris Assay

Prior to performing POC.auris assays, assay cartridges with pre-loaded reagents were prepared. Specifically, 49 μL wash buffer supplemented with 1 μL 10% Tween 20 and 20 μL PCR mix were loaded into the wash buffer well and the PCR well of an open cartridge, respectively. The cap layer was then capped onto the open cartridge *via* PSA before 450 μL silicone oil (50 cSt, Millipore-Sigma, USA) was injected through the sample injection opening to overlay both the wash buffer and the PCR mix. This immiscible silicone oil layer served both as a medium for transporting the magnetic beads in the cartridge and as a separator that prevented the mixing of assay reagents between the wells. The cartridge was either used immediately or placed on ice with the sample injection opening seal with tape (Scotch Magic Tape, 3M, USA) until use. Of note, these reagents added USD $0.98 per cartridge to the cost of each POC.auris assay (Supplementary Table S4).

For performing POC.auris, a 100-μL sample (e.g., *C. auris* lysate in either 1× PBS or ESwab^®^ medium along with 1 µL control DNA plasmid and 1 µL human genomic DNA for simulating background DNA that may be present in a swab sample) was pipette mixed with 22 μL magnetic bead buffer and then loaded into the sample well of the assay cartridge. After sample loading, the cartridge was tape-sealed (Scotch Magic Tape, 3M, USA) and mounted onto the faceplate of the DM device with the PCR mix well of the assay cartridge seated in the aluminum heat block, whose inner surface was coated with a thermally conductive paste (Arctic Silver 5, Visalia, California, USA) for ensuring consistent thermal contact throughout PCR. The faceplate was then magnetically clasped onto the main housing of the device, such that the sample well was positioned between the pair of permanent magnets of the motorized magnetic arm.

POC.auris began with a ∼3-min automated sample preparation process, during which the magnetic beads and bound DNA were concentrated from the sample well, transferred to the wash buffer well for wash, and finally transferred to the PCR mix well. Once the magnetic beads and bound DNA arrived in the PCR mix well, the thermocycling module began thermocycling with concurrent real-time fluorescence detection. PCR began with a 95°C hot start for 2 min followed by 45 cycles of 106°C for 1 s and 65°C for 15 s. The fluorescence detector measured the fluorescence signal emitted from the PCR mix at well every cycle at the annealing and extension step.

### Analytical Sensitivity and Analytical Specificity of POC.auris

*C. auris* AR0382 was used to assess the analytical sensitivity of POC.auris. Here, *C. auris* AR0382 cell suspension was initially adjusted to OD600 = 0.1 (∼3 × 10^6^ CFU/ml (Bravo Ruiz et al., 2019; Lima et al., 2019; Srivastava and Ahmad, 2020)) and then serially diluted in PBS to final concentrations of 3×10^5^, 3×10^4^, 3×10^3^, and 3 × 10^2^ CFU/ml. One hundred µL of each cell dilution was used as the sample. Each sample was subjected to 10 cycles of lysis by an OmniLyse device. All 100 µL of lysate was mixed with 22 µL magnetic bead buffer, 1 µL control DNA plasmid, and 1 µL of 3.6 μg/ml human genomic and subsequently, loaded into the sample well of the DM assay cartridge to initiate POC.auris. For evaluating the analytical specificity of POC.auris, we tested all 3 strains of *C. auris* along with *C. albicans*, *C. glabrata*, *C. parapsilosis*, and *C. tropicalis*. All *Candida* species cell suspensions were initially adjusted to OD600 = 0.1 (∼3 × 10^6^ CFU/ml (Bravo Ruiz et al., 2019; Lima et al., 2019; Srivastava and Ahmad, 2020)) and then diluted in PBS to final concentrations of 10^3^ CFU/ml. One hundred µL of each cell dilution was used as the sample. Each sample was subjected to 10 cycles of lysis by an OmniLyse device. All 100 µL of lysate was mixed with 22 µL magnetic bead buffer, 1 µL control DNA plasmid and 1 µL of 3.6 μg/ml human genomic DNA and subsequently loaded into the sample well of the DM assay cartridge to initiate POC.auris.

### Data Analysis and Presentation

Data were analyzed *via* Microsoft Excel 365 and plotted in Origin 2018. The fluorescence signals and C_q_ values measured by the Bio-Rad CFX96 system (Supplementary Figures S3–S7, S9) were first exported to Excel and subsequently plotted in Origin. For POC.auris, the FAM and TYE fluorescence signals measured by the fluorescence detector in the droplet magnetofluidic device (Supplementary Figures S8, S10–S13) were first exported to Excel. In Excel, both FAM and TYE fluorescence signals of the first PCR cycle were subtracted from FAM and TYE fluorescence signals of each PCR cycle. The subtracted FAM and TYE fluorescence signals were subsequently plotted in Origin. POC.auris C_q_ values were directly calculated by the linear regression algorithm that was built-in companion software of DM device. For evaluating the analytical sensitivity of POC.auris, the mean and standard deviation of C_q_ values from >3 technical replicates were calculated in Excel and subsequently were plotted in Origin, where the data were presented as mean ± 1SD.

## Results

### Overview of POC.auris

**FIGURE 1:**
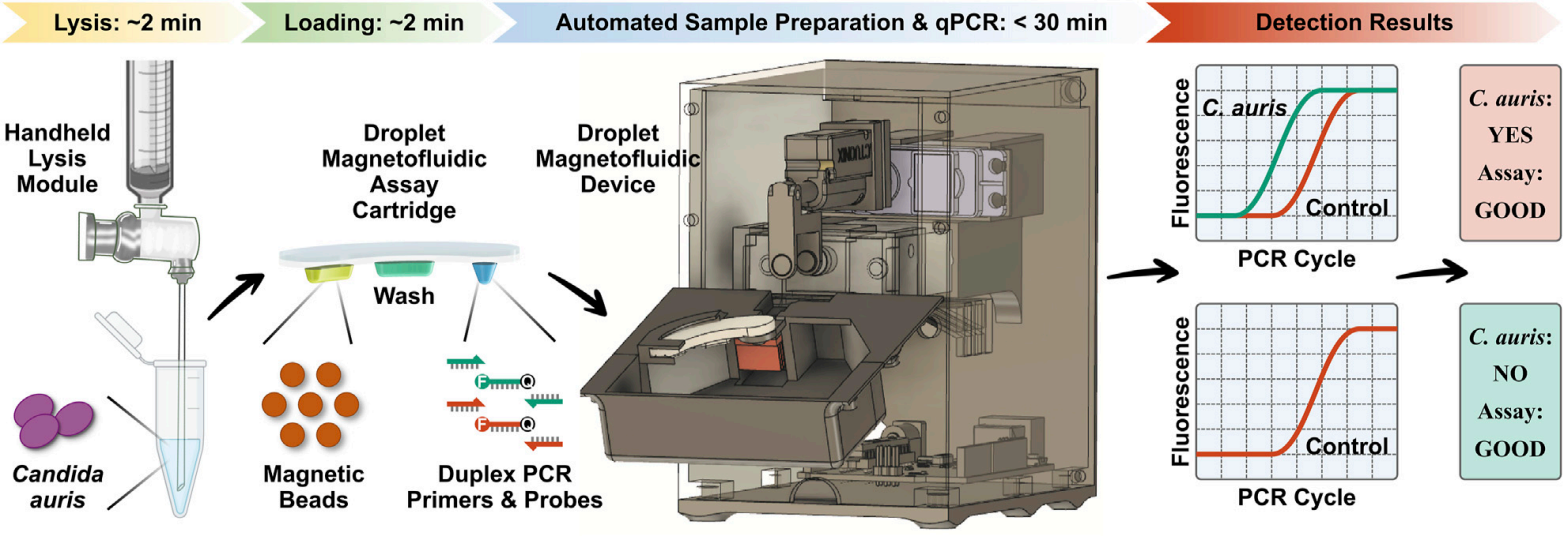
Overview of POC.auris—a rapid, streamlined, and POC-amenable diagnostic testing solution for *C. auris* with the aid of droplet magnetofluidic (DM) technology. POC.auris begins with handheld bead-beating-based lysis of *C. auris* cells that can lyse *C. auris* cells with ∼2 min hands-on time. The lysate, a magnetic bead buffer, and an internal control DNA plasmid are loaded into the sample well of the DM assay cartridge, where DNA is captured by the magnetic beads. The assay cartridge also contains a wash buffer well for purifying DNA and a PCR mixture well, which holds PCR primers and fluorophore (F)- and quencher (Q)-labeled TaqMan probes for amplifying and detecting *C. auris* and the internal control. After the ∼2 min loading step, the assay cartridge is inserted in the DM device to initiate the assay in full automation. Within the device, DNA is magnetically transported to the wash buffer and to the PCR mixture, where DNA is amplified and detected *via* rapid duplex real-time quantitative PCR (qPCR). The resulting PCR curves for *C. auris* (green) and the internal control (red), which can be acquired in <30 min, indicate the presence or absence of *C. auris* and the reliability of the assay.

POC.auris provides a rapid, streamlined, and POC-amenable diagnostic testing solution for *C. auris* with the aid of DM (Figure 1). The assay begins with lysis of *C. auris* cells *via* a handheld bead-beating-based lysis module that can effectively lyse *C. auris* cells with ∼2 min hands-on time. The lysate is loaded along with a magnetic bead buffer and an internal control DNA plasmid into the sample well of the DM assay cartridge, where DNA is captured by the magnetic beads. The assay cartridge also houses a wash buffer for DNA purification and a PCR mixture that contains primers and TaqMan probes for amplification and detection of *C. auris* and the internal control. After the ∼2 min loading step, the assay cartridge is inserted in the DM device, and the device is prompted to initiate the automated assay by first magnetically transporting the magnetic bead pellet and the captured DNA from the sample well to the wash buffer and then to the PCR mixture. Once DNA arrives in the PCR mixture well, the device commences duplex qPCR with concurrent dual-color fluorescence detection after every PCR cycle. Importantly, as the DM device employs a miniaturized thermocycling module, the duplex qPCR assay is significantly accelerated, and the turnaround time for the entire automated assay is shortened to <30 min. Finally, the resulting PCR curves for *C. auris* and the internal control indicate the presence or absence of *C. auris* and the reliability of the assay.

POC.auris employs our previously reported disposable thermoplastic DM assay cartridge (Supplementary Figure S1) and the integrated portable DM device (Supplementary Figure S2) (Chen F.-E. et al., 2021; Chen L. et al., 2021; Trick Alexander et al., 2021). The assay cartridge has a compact footprint (similar to a USB drive) and is fabricated *via* simple assembly of low-cost plastic materials. The assay cartridge has 3 independent wells for holding the mixture of sample and magnetic bead buffer (*pH* = 5), a wash buffer (*pH* = 7), and the PCR mix, which are further separated by an immiscible oil that prevents evaporation and mixing of these aqueous reagents. After sample loading, each disposable assay cartridge is tape-sealed and mounted in the portable DM device. The device houses a motorized magnetic arm, a miniature thermocycling module, a compact dual-color fluorescence detector, and a microcontroller for automating these components. The motorized magnetic arm, which is equipped with a pair of permanent magnets located at the opposing ends of the assay cartridge, moves bi-axially to concentrate, and sequentially transport the magnetic beads from the sample well to the wash buffer well and then to the PCR reaction mix well. Upon the arrival of the magnetic beads and DNA, the thermocycling module begins to thermocycle the PCR reaction mix well while the fluorescence detector measures the fluorescence emitted from PCR in real time.

### Development of Benchtop Droplet Magnetofluidic-Compatible Rapid Duplex qPCR

**FIGURE 2:**
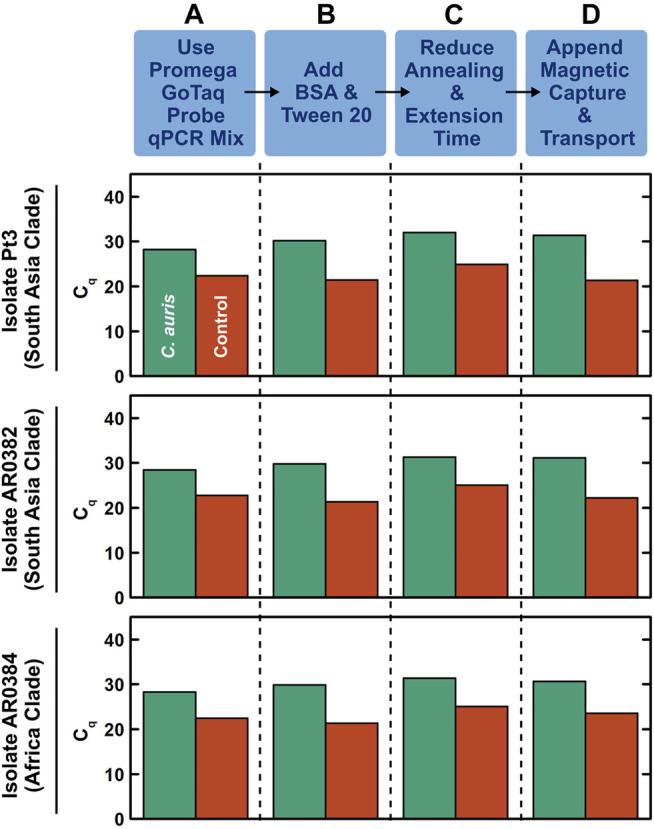
Development of benchtop droplet magnetofluidic-compatible rapid duplex qPCR. The step-by-step development process shown here employs benchtop extracted and purified DNA (100 fg) from 3 strains of *C. auris* that originate from 2 major clades (South Asia and Africa) and a control DNA plasmid containing the bicoid gene (10 pg) as the inputs. **(A)** Using Promega GoTaq Probe qPCR Master Mix, which is compatible with droplet magnetofluidics, under its fast cycling protocol (95°C hot start for 2 min and 40 cycles of 95°C for 3 s and 60°C for 30 s), both *C. auris* DNA and control DNA plasmid directly spiked into the PCR mix can be detected, as indicated by clear cycle of quantification (C_q_) values in both the FAM channel associated with the *C. auris* probe (green) and the TYE channel associated with the bicoid probe (red). The C_q_ values are comparable among the 3 strains of *C. auris*. **(B)** Addition of BSA and Tween 20—standard additives for PCR in droplet magnetofluidic assays—causes negligible differences to the PCR assay. **(C)** Accelerating the PCR assay by reducing the annealing and extension step at 60°C from 30 to 15 s only slightly delays the C_q_ values. **(D)** Appending manual magnetic capture and transport of both *C. auris* DNA and control DNA plasmid upstream to the accelerated rapid duplex qPCR assay still result in successful detection of both targets. This assay can be readily adapted into the droplet magnetofluidic cartridge and device.

We first developed a benchtop rapid duplex qPCR assay that is compatible with DM. To do so, we employed US CDC-adopted primers and probes that target the ITS2 region of *C. auris* and an internal control plasmid (Leach et al., 2018), and verified their compatibility with rapid PCR and DM in a step-by-step process. In these initial developments, we used benchtop extracted and purified DNA from 3 clinically isolated *C. auris* that originate from 2 major clades (South Asia and Africa) and a control DNA plasmid containing the bicoid gene. We tested 1 ng, 10 pg, and 100 fg purified *C. auris* genomic DNA (corresponding to ∼70000, ∼700, and ∼7 copies) and 10 pg control DNA plasmid. We first verified that both *C. auris* DNA and bicoid plasmid DNA could be detected using one of our DM-compatible PCR mixes (Promega GoTaq Probe qPCR Master Mix) under its fast cycling protocol with 95°C hot start for 2 min and 40 cycles of 95°C for 3 s and 60°C for 30 s, as indicated by robust PCR curves (Supplementary Figure S3) and clear cycle of quantification (C_q_) values in both the FAM channel associated with the *C. auris* probe and the TYE channel associated with the bicoid probe (Figure 2A, green and red, respectively). We subsequently ensured the addition of BSA and Tween 20—standard PCR additives in our magnetofluidic assays—causes negligible differences to the PCR assay (Figure 2B and Supplementary Figure S4). We further accelerated the benchtop PCR by reducing the annealing and extension step at 60°C from 30 to 15 s, which still retained comparable amplification efficiency (Figure 2C and Supplementary Figure S5). We next verified the specificity of the *C. auris* primers by testing against 7 other *Candida* species (Suppplementary Figure S6). Notably, the primers successfully discriminated against *C. haemulonii* and *C. duobushaemulonii*, which are genetically close to *C. auris*. Finally, to simulate clinical swab eluates, we added human genomic DNA as background DNA to the sample before appending manual magnetic bead-based DNA capture upstream to rapid duplex qPCR. Even with the added background of human genomic DNA, we successfully detected 100 fg *C. auris* DNA and 10 pg control DNA (Figure 2D). In the absence of *C. auris* DNA, we still detected the control DNA plasmid, which effectively illustrates its control function (Supplementary Figure S7). Importantly, the C_q_ values for the samples with magnetically extracted DNA were comparable to those for the samples with directly spiked DNA (Supplementary Figure S7), which suggests sufficient recovery of DNA in our assay. These results establish our benchtop DM-compatible rapid duplex qPCR assay for detecting *C. auris* with assay control.

### Development of POC.auris With Handheld Cell Lysis

**FIGURE 3:**
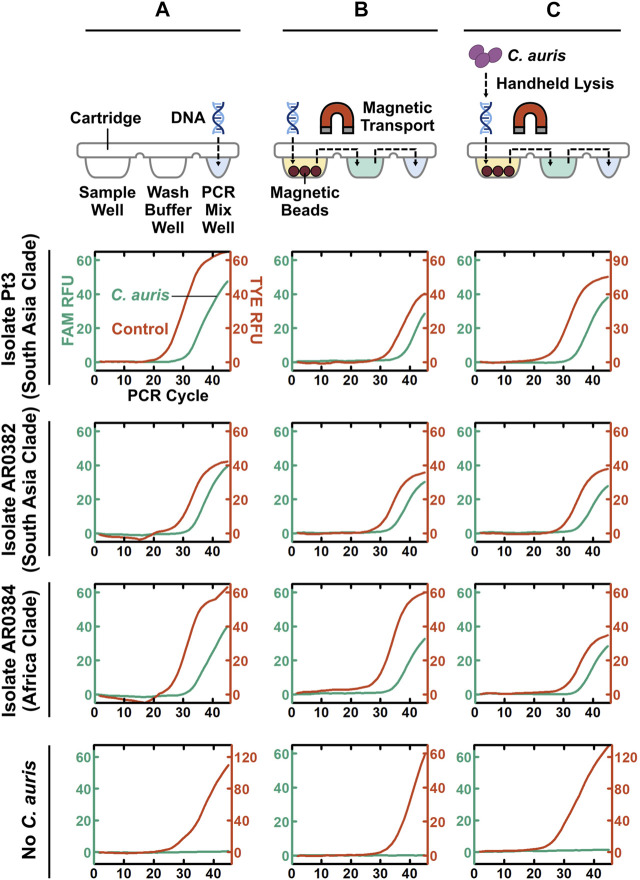
Step-by-step development of POC.auris. **(A)** Benchtop purified *C. auris* DNA from 2 major clades (100 fg) and the control DNA plasmid (1 pg), as well as the no-*C. auris* DNA control, are directly spiked into the PCR mix well of assay cartridges, which are then mounted in the DM device to perform rapid duplex qPCR. Robust *C. auris* curves (green) and control DNA plasmid curves (red) indicate successful duplex qPCR performed by the miniature thermocycling module and the fluorescence detector in the DM device. **(B)** Benchtop purified *C. auris* DNA from 2 major clades (100 fg) and the control DNA plasmid (1 pg), as well as the no-*C. auris* DNA control, are first loaded into the sample well of DM assay cartridges. Subsequently, within the DM device, both *C. auris* DNA and control DNA plasmid are captured by magnetic beads, transported through the wash buffer well to the PCR mix well, eluted directly in PCR mix, and amplified and detection *via* rapid duplex qPCR—all in full automation. Robust *C. auris* PCR curves (green) and control DNA plasmid PCR curves (red) demonstrate successful DNA extraction, transport, and elution within the assay cartridges and the device. **(C)** A simple, fast, and handheld method for lysing *C. auris* cells is incorporated to complete the development of POC.auris. This lysis method uses a single-use microbead-beating module that is battery-powered to mechanically lyse *C. auris* cells. *C. auris* cells from 2 major clades at 3 × 10^3^ CFU/ml serve as samples and are subjected to full POC.auris—including handheld cell lysis, automated magnetic-based DNA extraction and transport, and rapid duplex qPCR. Robust *C. auris* PCR curves (green) demonstrate that all 3 strains are detected by POC.auris.

Taking a step-by-step approach for establishing POC.auris, we first adapted the benchtop rapid duplex qPCR within the DM assay cartridge and the portable DM device. Here, we directly spiked benchtop which was extracted and purified *C. auris* DNA and the internal control DNA plasmid in the PCR well of the assay cartridge and performed PCR using the miniature thermocycling module and the fluorescence detector in the DM device. We adopted the elevated denaturation temperature (106°C) and the short denaturation time per PCR cycle (1 s) used in the DM device from our earlier works (Chen L. et al., 2021; Trick Alexander et al., 2021); this denaturation condition was previously verified to support PCR without causing visible evaporation of the PCR mix. Combined with 15 s annealing and extension time per PCR cycle, the duplex qPCR assay with 45 cycles could be completed in ∼26 min in the DM. We subsequently fine-tuned the annealing and extension temperature in the DM device to ensure robust duplex qPCR even with rapid thermocycling (Supplementary Figure S8). We found that using 65°C for annealing and extension, duplex qPCR in our assay cartridge and device could robustly detect 100 fg purified DNA from the 3 strains of *C. auris* and 1 pg control plasmid DNA, as indicated by clear PCR curves in both the FAM and TYE channels (Figure 3A). Of note, during the development of POC.auris, we switched the provider for the control DNA plasmid and found that 1 pg was sufficient for providing control in the absence of *C. auris* DNA (Figure 3A).

We subsequently appended magnetic bead-based capture and transport of DNA prior to PCR. Specifically, we loaded benchtop extracted and purified *C. auris* DNA, the internal control DNA plasmid, and human genomic DNA (as background DNA for simulating clinical swab elutes) along with magnetic bead buffer into the sample well of each assay cartridge and allowed binding between DNA and magnetic beads. We then immediately installed the assay cartridge in the DM device and initiated its automated sample preparation and PCR protocol. Within the device, the motorized magnetic arm concentrated the DNA-bound magnetic beads into a pellet, transported it through the wash buffer well, and finally was delivered it to the PCR mix well (Figure 3B). In the PCR mix well, DNA dissociated from the beads before the beads were removed from the PCR mix well and PCR commenced. We were able to simultaneously detect 100 fg purified DNA from the 3 strains of *C. auris* and the control DNA plasmid, as well as only the control DNA plasmid in the absence of *C. auris* DNA (Figure 3B). Of note, the C_q_ values of the three magnetically extracted *C. auris* DNA samples were ∼3 cycles higher than their directly spiked counterparts, suggesting ∼10% efficiency in DNA capture and transport. Nevertheless, these results demonstrate that DNA was magnetically captured and transported through the cartridge and directly eluted in the PCR mix.

As the final step of POC.auris development, we incorporated a simple, fast, and handheld method for lysing *C. auris* cells. To use this method, we employed a syringe-linked microbead-beating module that is battery-powered and disposable to mechanically lyse *C. auris* cells (Supplementary Figure S9) (Vandeventer Peter et al., 2011; Shin et al., 2017). For characterizing this method, we spiked 3 × 10^6^ CFU/mL *C. auris* cells, which were cultured from the isolate from the South Asia clade, into PBS as the sample. We determined that 10 lysis cycles (i.e., passing the sample through the lysis module 10 times), which were completed in ∼1—2 min, achieved effective lysis of *C. auris* (Supplementary Figure S9). To our knowledge, these results represent the first demonstration of such handheld lysis of *C. auris*.

We demonstrated the utility of full POC.auris—including handheld cell lysis, automated magnetic sample preparation, and rapid duplex qPCR—with multiple strains of *C. auris*. Here, we first challenged POC.auris against 5 strains of *C. auris*, which belong to the South Asia clade, the Africa clade, and the South America clade, in PBS suspension at ∼3 × 10^3^ CFU/ml, and we included a no-*C. auris* control (i.e., only PBS). After subjecting each sample and the control with 10 lysis cycles, we loaded the lysate along with the control DNA plasmid, human genomic DNA, and the magnetic bead buffer into the sample well of an assay cartridge and initiated our workflow. We were able to detect all 5 strains of *C. auris* along with their respective control DNA plasmid, and only the control DNA plasmid from the no-*C. auris* control (Figure 3C and Supplementary Figure S10). Moreover, we were able to detect *C. auris* strain from the South America clade suspended in a swab transport medium (Supplementary Figure S11). These results demonstrate that POC.auris could detect up to 5 strains of *C. auris* belonging to 3 major clades from either PBS or a swab transport medium.

### Analytical Performance Evaluation of POC.auris

**FIGURE 4:**
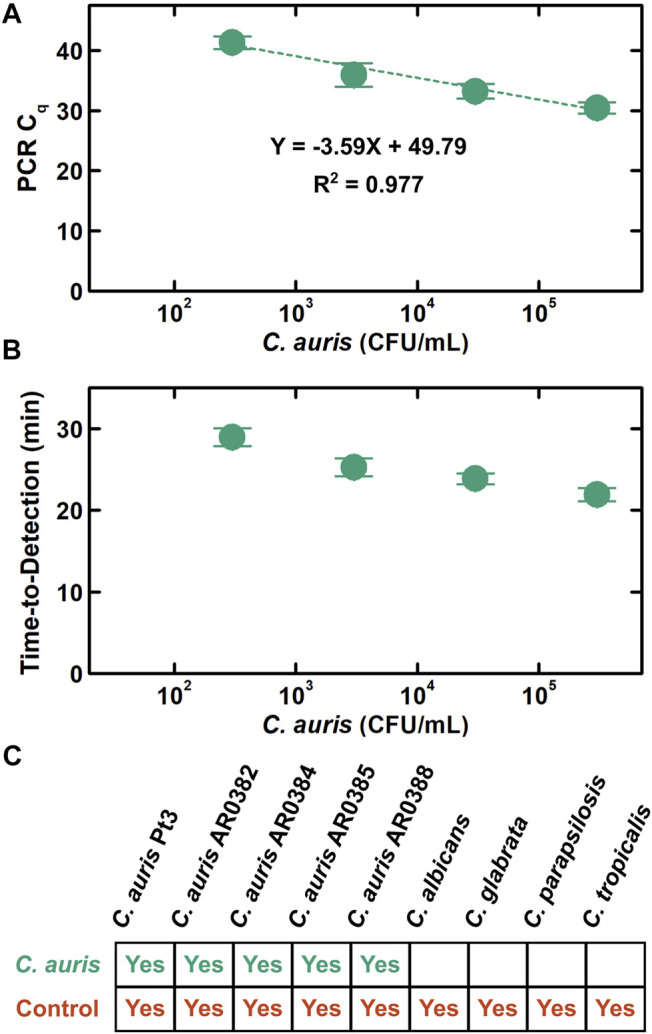
Analytical performance evaluation of POC.auris. **(A)** POC.auris detects 3×10^5^, 3×10^4^, 3×10^3^, and 3 × 10^2^ CFU/ml of *C. auris* strain 0382 (South Asia clade) in PBS with C_q_ values of 30.5 ± 1.0, 33.2 ± 1.2, 35.9 ± 2.0, and 41.3 ± 1.0, respectively (mean ±1 SD, n ≥ 3). The slope (−3.59) of the linear fit (*R* ^2^ = 0.977) suggests efficient PCR in the DM assay cartridge and the DM device. **(B)** For 3×10^5^, 3×10^4^, 3×10^3^, and 3 × 10^2^ CFU/ml of *C. auris* strain 0382, the time-to-detection in POC.auris—the elapsed time between initiating the automated program in the DM device and detecting *C. auris* through a definitive C_q_ value measured by the DM device—are 21.9 ± 0.8, 23.9 ± 0.7, 25.3 ± 1.1, and 28.9 ± 1.1 min, respectively (mean ±1 SD, n ≥ 3). **(C)** POC.auris specifically detects *C. auris*. When tested against 4 other *Candida* species including *C. albicans*, *C. glabrata*, *C. parapsilosis*, and *C. tropicalis*, POC.auris shows no cross reactivity and only detects the internal control DNA plasmid.

As part of analytical performance evaluation of POC.auris, we first assessed its analytical sensitivity. Here, we tested POC.auris against a 10-fold serial dilution series from 3×10^5^ to 3 × 10^2^ CFU/ml of *C. auris* cells cultured from the isolate of the South Asia clade and spiked into PBS. In these experiments, we continued employing PBS because PBS has been used as an elution and transport medium for *C. auris* swabs (Leach et al., 2018), and therefore is a clinically relevant medium. The C_q_ values for 3×10^5^, 3×10^4^, 3×10^3^, and 3 × 10^2^ CFU/ml measured by POC.auris were 30.5 ± 1.0, 33.2 ± 1.2, 35.9 ± 2.0, and 41.3 ± 1.0, respectively (mean ±1 SD; Figure 4A, n ≥ 3). We observed strong linearity from our results (*R* ^2^ = 0.977). The slope of our linear fit was −3.59, which corresponds to ∼90% efficiency. Finally, we note that these results were acquired on different days, the low coefficients of variation across the four target concentrations (all ≤5.4%; Supplementary Figure S12) thus demonstrate that POC.auris is reproducible.

To show the speed of POC.auris, we calculated the time-to-detection for each input concentration of *C. auris*. In this study, the time-to-detection represents the elapsed time between initiating the automated program in the DM device (i.e., immediately after mounting the DM assay cartridge in the DM device) and detecting *C. auris* through a definitive C_q_ value measured by the DM device. The time-to-detection thus includes the time for executing magnetic bead-based capture and transport of DNA. The time-to-detection values for 3×10^5^, 3×10^4^, 3×10^3^, and 3 × 10^2^ CFU/mL *C. auris* were 21.9 ± 0.8, 23.9 ± 0.7, 25.3 ± 1.1, and 28.9 ± 1.1 min, respectively (mean ±1 SD; Figure 4B, n ≥ 3). These results illustrate that POC.auris can rapidly detect even low concentrations of *C. auris*.

Finally, we verified the analytical specificity of POC.auris against other *Candida* species. To do so, we cultured clinically isolated *C. albicans*, *C. glabrata*, *C. parapsilosis*, and *C. tropicalis* and tested POC.auris with these 4 species at 3 × 10^3^ CFU/ml. These *Candida* species were undetected by the *C. auris* probe (Figure 4C and Supplementary Figure S13), which supported that POC.auris is specific for detecting *C. auris*. Importantly, the internal control DNA plasmids mixed with these *Candida* species could still be detected, which ascertained the functionality of the assay and illustrated the significance of the internal control in POC.auris.

## Discussion

We have developed POC.auris—an automated and portable DM device that detects *C. auris* with fast speed, high sensitivity, and high specificity. For developing POC.auris, we first employed a US CDC-approved duplex qPCR assay that detects *C. auris* and an assay control and we systematically modified the assay to ensure its compatibility with DM. The modifications include using a DM-compatible PCR mix, incorporating additives, shortening the assay time, and appending magnetic-based DNA purification upstream. We subsequently adapted the DM-compatible assay into the disposable thermoplastic DM cartridge and the integrated palm-sized DM device to actualize POC.auris. We established that the miniature thermocycling module and the portable fluorescence detector in the device facilitated rapid duplex qPCR, then verified that the motorized magnetic arm in the device automated magnetic DNA capture and transport across the assay cartridge, and finally incorporated a handheld, bead-beating-based cell lysis module that lysed *C. auris* cells with <2 min hands-on time. Using POC.auris, we can detect clinically isolated *C. auris* from 3 major clades in <30 min, with no cross reactivity against other *Candida* species and LOD of ∼300 CFU/ml.

We made a few remarks to contextualize our work. First, while benchtop nucleic acid-based *C. auris* detection assays have been reported, POC.auris is unique because we have adapted our assay in our POC-amenable device. A recent review on *C. auris* diagnostics (Dennis et al., 2021) corroborates the lack of POC devices capable of nucleic acid-based detection of *C. auris*. Second, amid increasing popularity of isothermal nucleic acid amplification testing assays such as loop mediated isothermal amplification (LAMP), which has seen a report of benchtop LAMP assay for *C. auris* detection (Yamamoto et al., 2018), it is worth noting that miniaturized and rapid qPCR within POC.auris already delivers portability, speed, and simplicity through automation—potential “advantages” that isothermal assays promise to deliver. Moreover, isothermal assays still require sample preparation before testing, whereas POC.auris already integrates sample preparation. Third, although DM has been previously leveraged to develop POC diagnostic devices for other pathogens, POC.auris presents an advantage by incorporating an internal assay control, which represents an important benefit for POC diagnostic devices. Finally, throughout the development of POC.auris, we evaluated its sensitivity, specificity, hands-on time, turnaround time, and cost. These performance metrics should serve as the basis of comparison when future *C. auris* POC diagnostic devices are developed.

We envision several improvements for the current version of POC.auris to bring it a step closer to monitoring patients, screening close patient contacts, and testing environmental sources. First, isolates suspended in either PBS or a swab transport medium along with background human DNA were used as mock samples for the development and demonstration of POC.auris. A more extensive investigation into different sample types (e.g., clinical swabs and environmental sponges) with a range of sample volumes by adjusting the magnetic bead buffer and the sample well capacity of the assay cartridge is critical to assessing clinically relevant sensitivity and hence the clinical utility of POC.auris. Second, the current POC.auris involves brief but manual off-cartridge cell lysis and cartridge preparation. A simpler workflow using an improved assay cartridge that performs in-cartridge cell lysis and preloads all necessary reagents is critical to deploying POC.auris at POC and alleviating potential biosafety concerns from handling *C. auris*. Third, the current POC.auris can detect ∼300 CFU/mL *C. auris* in <30 min. Enhancing the sensitivity and the turnaround time by further optimizing DNA extraction efficiency and accelerating PCR in the DM device may improve its usefulness at POC. Fourth, the current POC.auris employed a qPCR assay that focuses on detecting *C. auris*, but different PCR assays may be adapted toward detecting antifungal resistance gene markers (Hou et al., 2019; Kordalewska et al., 2019) or differentiating *C. auris* strains. Finally, both the one-time cost of the DM device and the cost per POC.auris assay can be reduced by employing inexpensive optical components such as a light-emitting diode (LED) and a complementary metal-oxide-semiconductor (CMOS) camera and exploring alternatives to the commercial cell lysis module. Based on the current performances and the potential for improvements, we envision a future version of POC.auris can become a useful diagnostic tool for combating *C. auris*.

## Data Availability

The original contributions presented in the study are included in the article/Supplementary Material, further inquiries can be directed to the corresponding authors.
