# Supplementary material for: A Portable Droplet Magnetofluidic Device for Point-of-Care Detection of Multidrug-Resistant Candida auris
Source: Front Bioeng Biotechnol. 2022 Mar 24;10:826694. doi: 10.3389/fbioe.2022.826694 (PMC9003015; doi:10.3389/fbioe.2022.826694)
Supplement: Supplementary file 1 [file DataSheet1.PDF]

Supplementary Material

A portable droplet magnetofluidic device for point-of-care detection of multi-drug resistant *Candida auris*

Pei-Wei Lee[1], Marissa Totten[2], Liben Chen[1], Fan-En Chen[3], Alexander Y. Trick[3], Kushagra Shah[3], Hoan Thanh Ngo[1], Mei Jin[3], Kuangwen Hsieh[3,*], Sean X. Zhang[2,*], Tza-Huei Wang[1,3,4,*]

[1]Department of Mechanical Engineering, Johns Hopkins University, Baltimore, MD, USA

[2]Division of Microbiology, Department of Pathology, Johns Hopkins School of Medicine, Baltimore, MD, USA

[3]Department of Biomedical Engineering, Johns Hopkins University, Baltimore, MD, USA

[4]Institute of NanoBioTechnology, Johns Hopkins University, Baltimore, MD, USA

*** Correspondence:**
Co-Corresponding Authors
khsieh4@jhu.edu, szhang28@jhmi.edu, thwang@jhu.edu

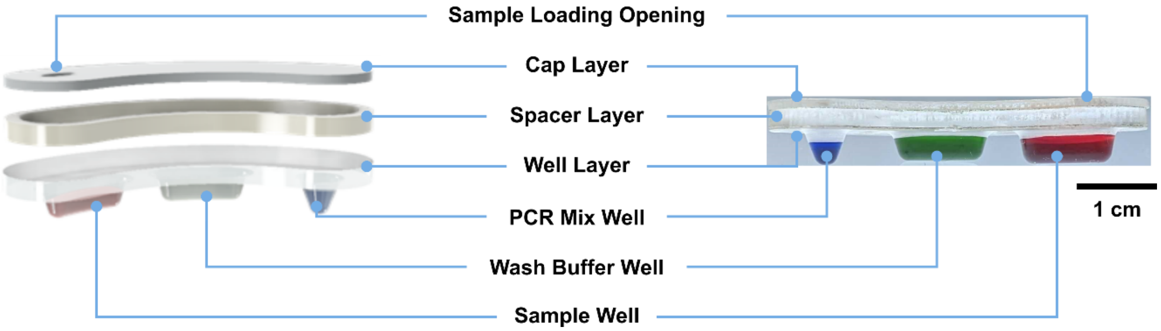

Fig. S1. Schematic and photograph of the droplet magnetofluidic assay cartridge. A schematic (left) and a photograph (right) of the droplet magnetofluidic assay cartridge together show the 3 layers – the top cap layer, the center spacer layer, and the bottom well layer – and the 3 wells in the well layer – the sample well, the wash buffer well, and the PCR mix well. The cap layer has a sample loading opening but encloses the rest of the cartridge and is made from laser-cut polymethylmethacrylate (PMMA) laminated with polytetrafluoroethylene (PTFE) tape. The spacer layer joins the layers and is made from laser-cut PMMA laminated with pressure-adhesive tape on both sides. The well layer holds the assay reagents and is made from thermoformed polypropylene. The individual layers are assembled to form the complete assay cartridge, which measures approximately 4 cm (L) × 1 cm (W) × 1 cm (H).

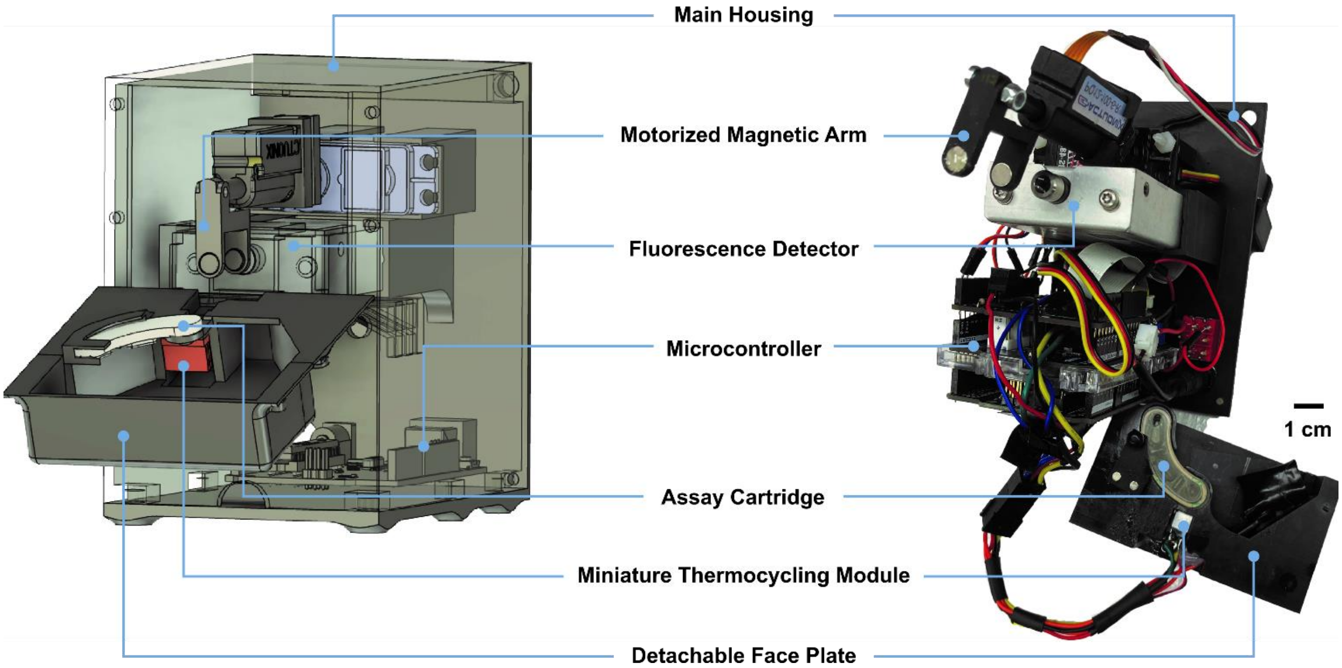

Fig. S2. Schematic and photograph of the droplet magnetofluidic device. A schematic (left) and a photograph (right) of the droplet magnetofluidic device together show the key device components, including the main housing, the motorized magnetic arm, the fluorescence detector, the microcontroller, the assay cartridge, the miniature thermocycling module, and the detachable faceplate. In the photograph, for clarity consideration, only the backside of the main housing is shown. In the schematic, the Arduino microcontroller is partially displayed. In the photograph, the full microcontroller that includes the motorshield and the custom printed circuit board is shown. The fully assembled device measures approximately 13.4 cm (L) × 8.4 cm (W) × 12.7 cm (H).

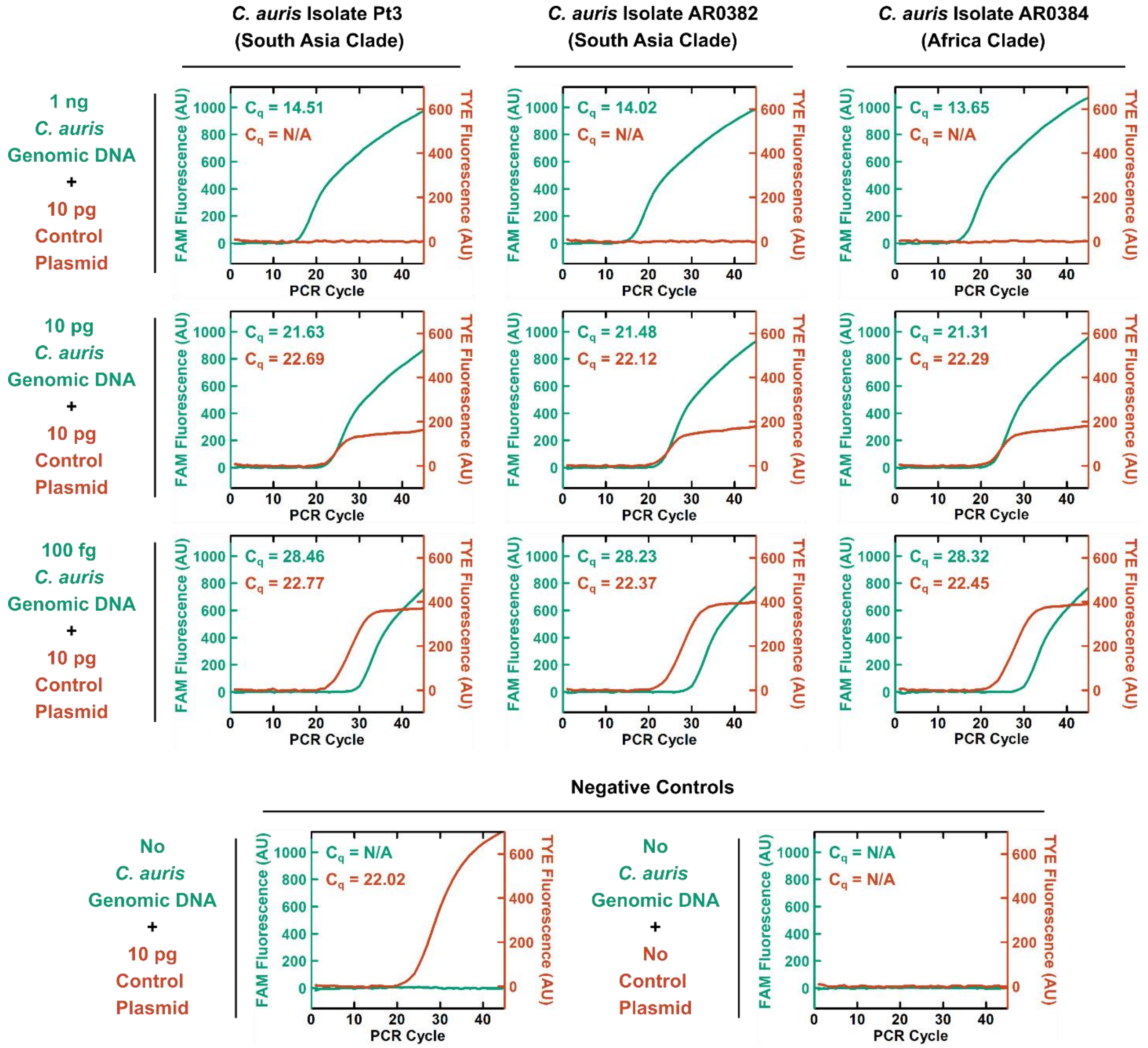

Fig. S3. Detection of *C. auris* DNA and control DNA via benchtop rapid duplex qPCR using US CDC-adopted primers and probes in droplet magnetofluidic-compatible PCR mix under its fast cycling condition. The PCR primers and probes, which have been previously reported and adopted by the US CDC, target the ITS2 region of *C. auris* and the bicoid gene region inserted within a DNA plasmid, which serves as the assay control. These PCR primers and probes are added to Promega GoTaq Probe qPCR Master Mix, which is compatible with droplet magnetofluidics. These primers and probes, and GoTaq Probe qPCR Master Mix are used throughout this work. The fast cycling condition entails a 95 °C hot start for 2 min followed by 45 cycles of 95 °C for 3 s and 60 °C for 30 s. Benchtop extracted and purified genomic DNA from 3 strains of *C. auris* belonging to 2 major clades (South Asia and Africa) at 1 ng, 10 pg, and 100 fg, as well as 10 pg of the control DNA plasmid are used as inputs for this initial test. For the results shown in this and relevant subsequent experiments (Figs. S4 – S7), all benchtop PCR are performed in a Bio-Rad CFX96 Touch Real-Time PCR Detection System, and the fluorescence signals are measured every cycle. The fluorescence signals measured by the Bio-Rad CFX96 system are baseline subtracted via the built-in curve fit function in the CFX Manager Software and the PCR cycles of quantification (C_q) are determined by the built-in single threshold

algorithm in the CFX Manager Software. *C. auris* DNA from both clades and all concentrations yield robust PCR curves (green), which demonstrate the compatibility between GoTaq Probe qPCR Master Mix under its fast cycling condition with the US CDC-adopted primers and probes. Of note, as the concentration of *C. auris* DNA decreases, the bicoid PCR curves (red) have earlier C_q values and higher intensities, suggesting some competition between the two targets in this rapid duplex qPCR assay. At 1 ng *C. auris* DNA, 10 pg control DNA plasmid is unamplified and undetected. Nevertheless, as 1 ng *C. auris* DNA represents a high *C. auris* concentration, a robust *C. auris* PCR curve without an accompanying bicoid PCR curve can still represent detection of *C. auris*.

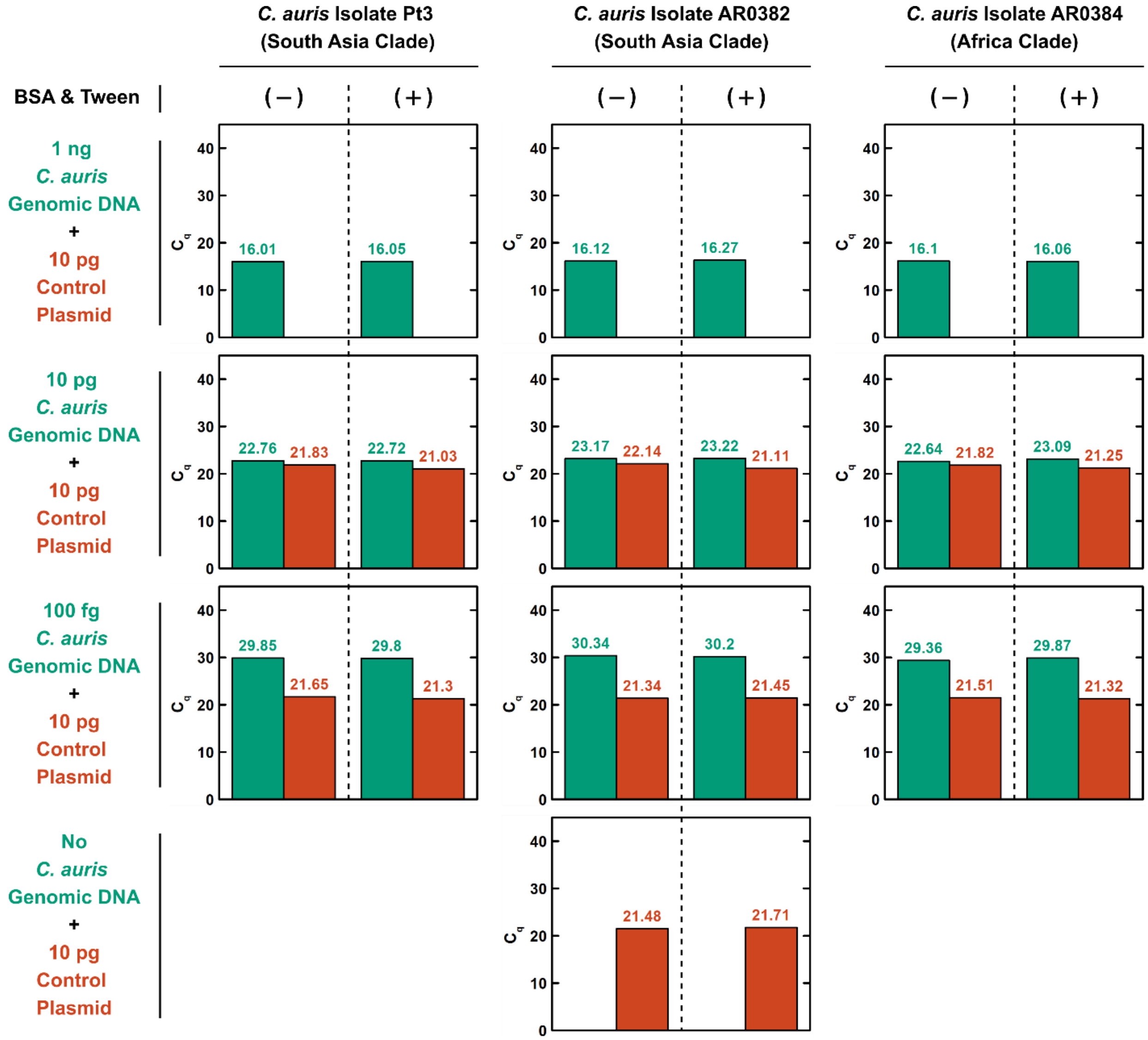

Fig. S4. Addition of bovine serum albumin (BSA) and Tween 20 to benchtop rapid duplex qPCR. For PCR reactions performed as part of droplet magnetofluidic assays, BSA and Tween 20 are required as additives. Therefore, for the development of benchtop droplet magnetofluidic-compatible rapid duplex qPCR, reactions with and without BSA (1 mg/mL) and Tween 20 (0.05%) are compared. The addition of BSA and Tween 20 causes negligible differences to the PCR assay across *C. auris* DNA (green bars) from both clades and all concentrations, as well as the control plasmids (red bars) and the no *C. auris* DNA controls, as indicated by comparable C_q values between the two sets of PCR. Based on these results, all subsequent rapid duplex qPCR are supplemented with 1 mg/mL BSA and 0.05% Tween 20.

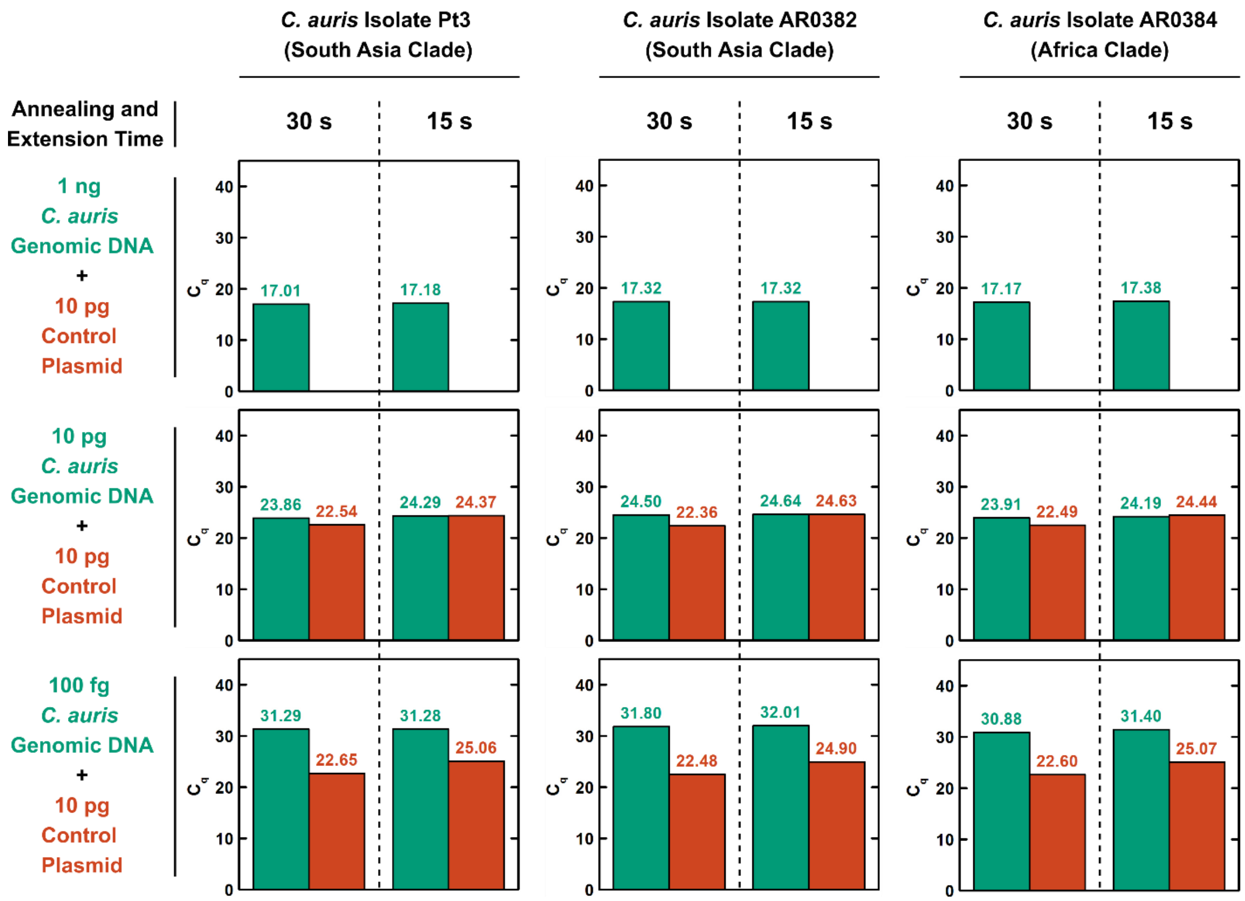

Fig. S5. Acceleration of benchtop rapid duplex qPCR. For the development of benchtop droplet magnetofluidic-compatible rapid duplex qPCR, the turnaround time can be accelerated by shortening the annealing and extension step (performed at a single temperature of 60 °C) of every PCR cycle from 30 s to 15 s. The shortened annealing and extension time causes negligible differences for detecting *C. auris* DNA (green bars) across both clades and all concentrations, as indicated by comparable C_q values between the two sets of PCR. With the shortened annealing and extension time, the control plasmids (red bars) are reliably detected in the presence of 10 pg and 100 fg *C. auris* DNA from both clades – consistent with prior results. However, the shortened annealing and extension time causes delays in the C_q values by ~2.5 cycles. Nevertheless, as the control function of the assay remains unaffected by the C_q delay, all subsequent rapid duplex qPCR employ 15 s annealing and extension of every PCR cycle.

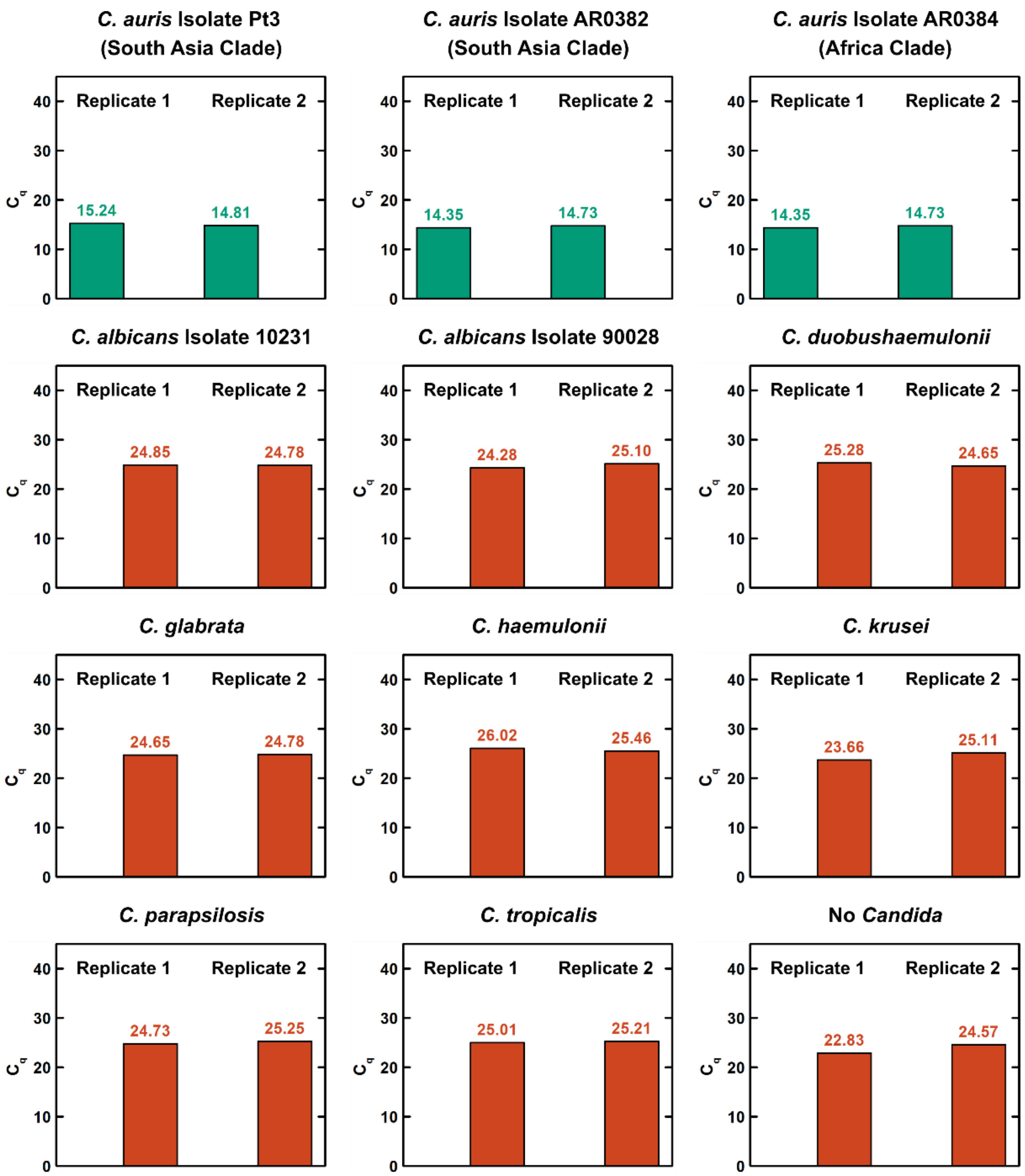

Fig. S6. Specificity of benchtop rapid duplex qPCR. Seven non-*C. auris Candida* species, including *C. albicans* (2 isolates), *C. duobushaemulonii*, *C. glabrata*, *C. haemulonii*, *C. krusei*, *C. parapsilosis*, and *C. tropicalis* are tested to verify the specificity of the benchtop rapid duplex qPCR. Here, genomic DNA from these *Candida* species are extracted and purified on benchtop, and 1 ng genomic DNA from each species and 10 pg control plasmid is spiked into the PCR mix. For comparison, 1 ng *C. auris* genomic DNA from both clades and a no *Candida* control, each of which includes 10 pg control plasmid, are performed in parallel. Of note, rather than using a low DNA concentration, a high concentration of 1 ng genomic DNA is deemed as a more appropriate test for specificity. All reactions are duplicated. As expected, *C. auris* DNA (green bars) from both clades are detected with undetectable control plasmid – consistent with prior results. For all non-C.auris *Candida* species and the no *Candida* control, only the control plasmids (red bars) are detected. These results confirm that the benchtop rapid duplex qPCR specifically detects *C. auris* DNA and that the control plasmid in the assay provides adequate assay control in the absence of *C. auris* DNA.

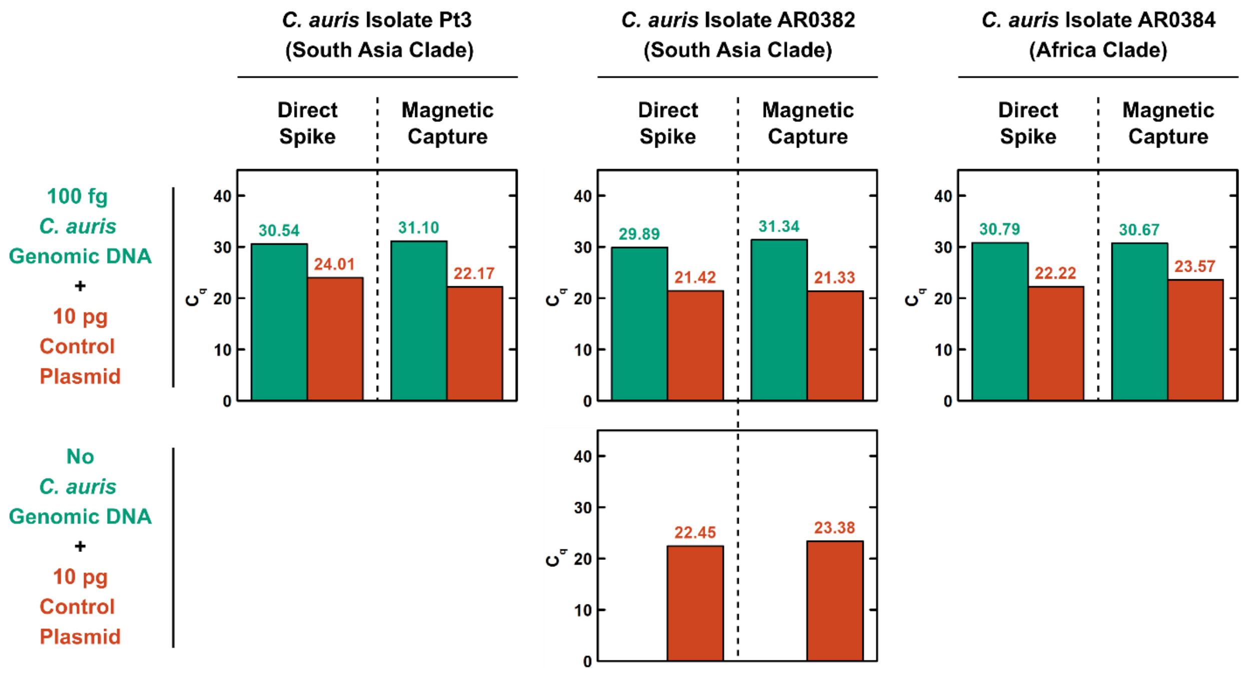

Fig. S7. Addition of magnetic-based DNA capture upstream to benchtop rapid duplex qPCR. To complete the development of benchtop droplet magnetofluidic-compatible rapid duplex qPCR, magnetic bead-based capture of DNA is added upstream to benchtop rapid duplex qPCR. ChargeSwitch™ gDNA Plant Kit, which can isolate genomic DNA from fungal samples, is employed here. Each 100 µL sample, which contains 1 µL 100 fg *C. auris* genomic DNA, 1 µL 10 pg control DNA plasmid, and 98 µL 1× PBS, is mixed with 14 µL magnetic bead buffer, which contains 4 µL ChargeSwitch magnetic particles and 10 µL Binding Buffer from ChargeSwitch™ gDNA Plant Kit. The mixture is kept at room temperature for 1 min to allow binding between magnetic beads and *C. auris* DNA and control DNA plasmid. A DynaMag™-2 Magnet is used to pellet the magnetic beads and bound DNA and facilitate washing with the Wash Buffer from ChargeSwitch™ gDNA Plant Kit. Ten µL rapid duplex qPCR mix is added to the pelleted magnetic beads in the tube to elute DNA directly in the PCR mix. Each PCR mix (along with eluted DNA) is transferred into a PCR tube. For comparison, 1 µL 100 fg *C. auris* genomic DNA and 1 µL 10 pg control DNA plasmid are directly spiked into 10 µL rapid duplex qPCR mix. All PCR are performed on benchtop in a Bio-Rad CFX96 Touch Real-Time PCR Detection System. Between the magnetically captured and the directly spiked samples, both *C. auris* DNA (green bars) and control plasmid (red bars) have comparable C_q values. These results suggest efficient magnetic-based capture, wash, and elution for both *C. auris* genomic DNA and control DNA plasmid and complete the development of benchtop droplet magnetofluidic-compatible rapid duplex qPCR.

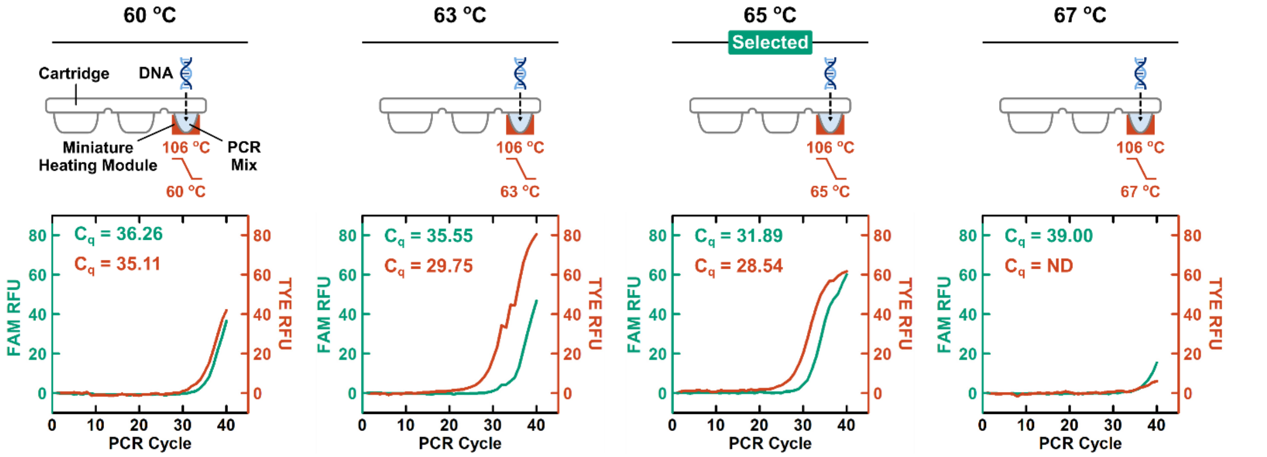

Fig. S8. Characterization of annealing and extension temperature for rapid duplex qPCR in droplet magnetofluidic assay cartridge and device. Ensuring robust rapid duplex qPCR in the droplet magnetofluidic assay cartridge and device represents a critical step for developing POC.auris. To this end, as the droplet magnetofluidic device employs a custom miniature thermocycling module that is different from the Bio-Rad CFX96 Touch Real-Time PCR Detection System, the PCR annealing and extension temperature is empirically tuned. Here, 100 fg *C. auris* genomic DNA (from isolate Pt3 that originates from India, which belongs to the South Asia clade) and 10 pg control DNA plasmid (from Addgene) are directly spiked into PCR mix and loaded into the PCR mix well of the assay cartridge. The assay cartridge is mounted in the device to commence rapid duplex qPCR. The denaturation temperature is set at 106 °C, which was empirically optimized in previous works. Rapid duplex qPCR begins with a 95 °C hot start for 2 min followed by 40 cycles of 106 °C for 1 s and either 60 °C, 63 °C, 65 °C, or 67 °C for 15 s. At 65 °C, both *C. auris* DNA (green curve) and control plasmid (red curve) have the earliest C_q values. 65 °C is therefore selected as the annealing and extension temperature for subsequent PCR conducted within the assay cartridge and the device.

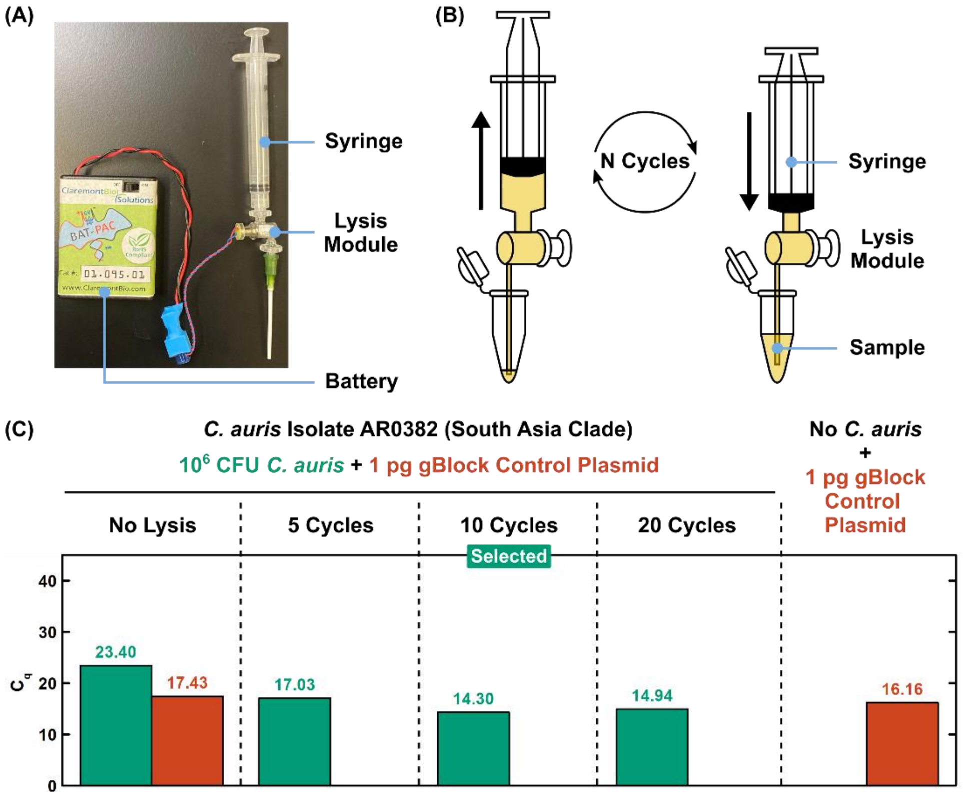

Fig. S9. Characterization of lysis of *C. auris* via the handheld lysis method. (A) The handheld lysis method employs commercial OmniLyse device, which is a single-use lysis module that performs micro-motor-based bead-beating mechanical lysis when connected to a single-use syringe and powered by a reusable battery. (B) To perform lysis, the sample is drawn and infused by the syringe through the powered-on lysis module for multiple cycles. Each cycle takes ~5 – 10 s. (C) Cell suspensions containing 10^6 CFU/mL *C. auris* isolate AR0382 (South Asia clade) in 1× PBS are used for characterizing this handheld lysis method. Three cell suspensions are used to determine an effective lysis cycle number. Each of these cell suspensions is paired with a lysis module (and a syringe), and subjected to either 5 cycles, 10 cycles, or 20 cycles of lysis. After lysis, 1 pg control plasmid (gBlock from IDT) is added to each lysate. For comparison, 1 pg control plasmid is also added to a cell suspension without lysis and a 1× PBS that serves as the no-*C. auris* control. These 5 samples are mixed with magnetic bead buffers and processed with benchtop magnetic-based DNA extraction and purification. Finally, DNA from each sample that is captured by the magnetic beads is eluted directly into rapid duplex qPCR mix, transferred into PCR tubes, and amplified and analyzed by benchtop rapid duplex qPCR (in a Bio-Rad CFX96 Touch Real-Time PCR Detection System). Even without lysis, *C. auris* DNA can be detected (green bar), which may be because the cell suspension contains a portion of dead *C. auris* cells and/or the magnetic bead buffer can chemically lyse some *C. auris* cells. Importantly, after 5, 10, and 20 cycles of lysis, *C. auris* DNA can be detected 6.37, 9.10, and 8.46 cycles earlier than the no lysis sample, respectively. The earlier C_q values illustrate that lysis allows more *C. auris* DNA to be detected. Of note, for these 3 samples, control plasmids are suppressed from amplification (i.e., absent red bars) due to the presence of more *C. auris* DNA when compared to the no lysis control. Among the 3 samples, 10 lysis cycles, which can be completed in < 2 min, are selected for subsequent experiments. Finally, as expected, only the control plasmid (red bar) is detected from the no-cell control.

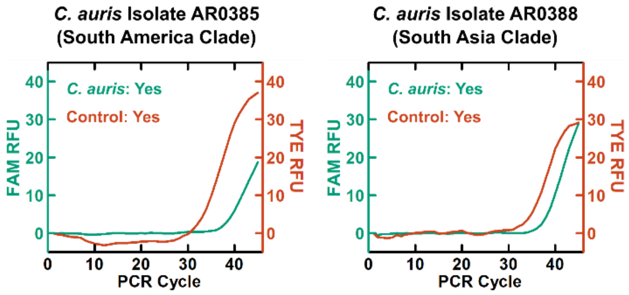

Fig. S10. Detection of additional *C. auris* strains via POC.auris. *C. auris* cells cultured from AR0385 isolate (South America clade) and AR0388 isolate (South Asia clade) and resuspended at ~3×10^3 CFU/mL in 1× PBS are used as samples and subjected to full POC.auris – including handheld cell lysis, automated magnetic-based DNA extraction and transport, and rapid duplex qPCR. Robust *C. auris* PCR curves (green) indicate that both strains are detected by POC.auris.

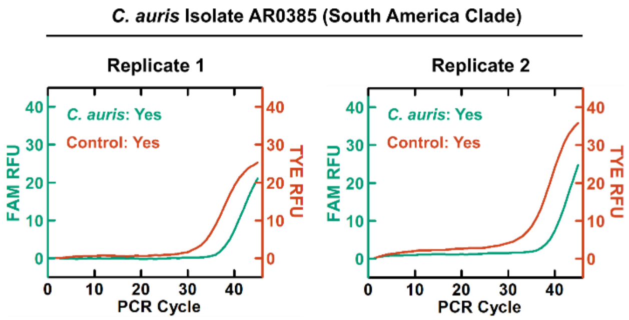

Fig. S11. Detection of *C. auris* in swab medium via POC.auris. *C. auris* cells cultured from AR0385 isolate (South America clade) and resuspended at ~3×10^3 CFU/mL in swab medium (ESwab® medium from Copan Diagnostics) are used as samples and subjected to full POC.auris. Robust *C. auris* PCR curves (green) in duplicate demonstrated that *C. auris* in swab medium can be repeatedly detected by POC.auris.

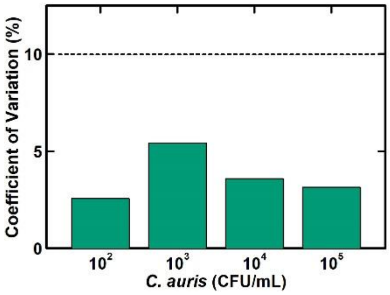

Fig. S12. Coefficient of variation (CV) from technical replicates using POC.auris. After using POC.auris to replicate ($n \geq 3$) in different days the detection of 10^5, 10^4, 10^3, and 10^2 CFU/mL of *C. auris* strain 0382 (South Asia clade) in PBS, the mean of C_q and the SD of C_q from each *C. auris* concentration are computed, and the CV is calculated by dividing the SD by the mean. The CV values from all 4 *C. auris* concentrations fall below 5.4%, which is lower than 10% (black dash line) – a common threshold for evaluating assay reproducibility. These low CV values thus support the reproducibility of POC.auris.

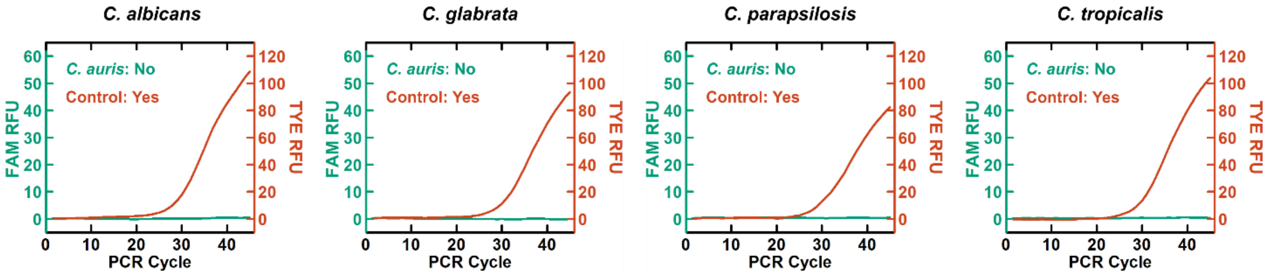

Fig. S13. PCR curves for non-*C. auris Candida* species acquired by POC.auris. For *C. albicans*, *C. glabrata*, *C. parapsilosis*, and *C. tropicalis*, POC.auris only detects the control DNA plasmid (red curves). These results illustrate the importance of the control plasmid in POC.auris and support the specificity of POC.auris.

Table S1. List of *Candida* species.

Species	Strain/Isolate	Source	Identification
C. auris	AR0382	U.S. Center for Disease Control and Prevention	N/A
C. auris	AR0384	U.S. Center for Disease Control and Prevention	N/A
C. auris	AR0385	U.S. Center for Disease Control and Prevention	N/A
C. auris	AR0388	U.S. Center for Disease Control and Prevention	N/A
C. auris	Clinical Isolate (Pt3)	Johns Hopkins Hospital Microbiology Laboratory	MALDI-TOF MS
C. albicans	Clinical Isolate	Johns Hopkins Hospital Microbiology Laboratory	MALDI-TOF MS
C. albicans	Clinical Isolate	Johns Hopkins Hospital Microbiology Laboratory	MALDI-TOF MS
C. duobushaemulonii	Clinical Isolate	Johns Hopkins Hospital Microbiology Laboratory	MALDI-TOF MS
C. glabrata	Clinical Isolate	Johns Hopkins Hospital Microbiology Laboratory	MALDI-TOF MS
C. haemulonii	Clinical Isolate	Johns Hopkins Hospital Microbiology Laboratory	MALDI-TOF MS
C. krusei	Clinical Isolate	Johns Hopkins Hospital Microbiology Laboratory	MALDI-TOF MS
C. parapsilosis	Clinical Isolate	Johns Hopkins Hospital Microbiology Laboratory	MALDI-TOF MS
C. tropicalis	Clinical Isolate	Johns Hopkins Hospital Microbiology Laboratory	MALDI-TOF MS

Table S2. Material cost breakdown of the droplet magnetofluidic assay cartridge.

Component	Vendor	Amount Per Purchase		Unit Price (USD)	Amount Per Cartridge		Price Per Cartridge (USD)
		Value	Unit		Value	Unit	
Top Acrylic Cover	Eplastics	1.00E+00	Sheet	$22.40	1.25E-03	Sheet	$0.03
Middle Acrylic Spacer	McMaster	1.00E+00	Sheet	$5.49	8.93E-03	Sheet	$0.05
Polypropylene (Thermoformed Wells)	Amazon	2.00E+01	Folder	$15.59	3.13E-02	Folder	$0.02
Teflon Tape	McMaster	5.40E+01	Foot	$113.98	5.00E-02	Foot	$0.11
9472LE 3M Transfer Tape	3M	1.92E+02	Foot	$280.68	7.14E-02	Foot	$0.10
						**Total**	**$0.31**

Table S3. Material cost breakdown of the droplet magnetofluidic device.

**Module in Device**	**Component**	**Vendor**	**Unit Price (USD)**	**Unit Per Device**	**Price Per Device (USD)**
**Motorized Magnetic Arm**	Linear Servo	Actuonix	$70.00	1	$70.00
	Rotational Servo	Amazon	$18.77	1	$18.77
	Neodymium Magnets - thick	Magnet4less	$0.17	5	$0.85
	Neodymium Magnets - rods	Magnet4less	$0.88	5	$4.40
**Miniature Thermocycling Module**	Microfan	PTI Pelonis Tech	$16.83	1	$16.83
	18kΩ Resistor	Digikey	$0.10	1	$0.10
	35kΩ Resistor	Digikey	$0.10	1	$0.10
	14kΩ Resistor	Digikey	$0.10	3	$0.30
	BD139 Transistor	Digikey	$0.49	2	$0.98
	14kΩ Semitec Thermistor	Mouser	$16.00	1	$16.00
	Thermoelectric Cooler	Custom Thermoelectric	$37.75	1	$37.75
	Flexible Thermal Epoxy	Custom Thermoelectric	$45.00	0.2	$9.00
	Thermal Paste	Custom Thermoelectric	$22.50	0.2	$4.50
**Fluorescence Detector**	Fluo Sens Detector (FAM/Cy5)	Qiagen	$3124.80	1	$3124.80
**Microcontroller**	5V Power Supply	Adafruit	$14.95	1	$14.95
	Arduino Uno	Amazon	$10.90	1	$10.90
	Arduino Motor Shield Rev3	Amazon	$24.60	1	$24.60
	Custom PCB	Advanced Circuits	$33.00	1	$33.00
**Housing & Connection**	3D-Printer Resin	Formlabs	$149.00	1	$149.00
	Male Crimp Pins for Female Connector	Digikey	$0.14	1	$0.14
	Female Crimp Pins for Male Connector	Digikey	$0.14	1	$0.14
	2 Pin Terminal	Digikey	$1.15	2	$2.30
				**Total**	**$3539.41**

Table S4. Reagent cost breakdown of each POC.auris assay.

Component	Vendor	Amount Per Purchase		Unit Price (USD)	Amount Per Assay		Price Per Assay (USD)
		Value	Unit		Value	Unit	
OmniLyse Single-Use Module	Claremont BioSolutions	4.80E+01	Module	$540.00	1.00E+00	Module	$11.25
GoTaq Probe qPCR Master Mix	Promega	2.00E+00	mL	$98.02	5.00E-03	mL	$0.25
C. auris Forward Primer	IDT	2.50E+01	nmol	$15.00	5.00E-03	nmol	< $0.01
C. auris Reverse Primer	IDT	2.50E+01	nmol	$15.00	5.00E-03	nmol	< $0.01
C. auris FAM Probe	IDT	1.00E+02	nmol	$175.50	1.00E-03	nmol	< $0.01
Bicoid Forward Primer	IDT	2.50E+01	nmol	$15.00	1.00E-03	nmol	< $0.01
Bicoid Reverse Primer	IDT	2.50E+01	nmol	$15.00	1.00E-03	nmol	< $0.01
Bicoid TYE Probe	IDT	1.00E+02	nmol	$283.50	1.00E-03	nmol	< $0.01
Bicoid gblock DNA	IDT	2.50+02	ng	$80.10	1.00E-03	ng	< $0.01
Tween20	Millipore Sigma	5.00E+01	mL	$23.86	5.00E-06	mL	< $0.01
BSA	NEB	1.20E+01	mg	$32.00	1.00E-02	mg	$0.03
ChargeSwitch Kit	ThermoFisher Scientific	2.00E+03	μL Beads	$264.00	4.00E+00	μL Beads	$0.53
Silicone Oil	Millipore Sigma	2.50E+02	mL	$92.50	4.50E-01	mL	$0.17
						**Total**	**$12.23**
